# Effect of Iron on the Microstructure, Mechanical Properties, Corrosion Behavior, and Biocompatibility of Mechanically Alloyed Zn-3Ag Biodegradable Alloys

**DOI:** 10.3390/jfb16120435

**Published:** 2025-11-25

**Authors:** Ilker Emin Dag, Ebru Erdal, Mohsen Mhadhbi, Baris Avar

**Affiliations:** 1 Department of Nanotechnology Engineering, Zonguldak Bülent Ecevit University, Zonguldak 67100, Türkiye; 2 Department of Metallurgical and Materials Engineering, Zonguldak Bülent Ecevit University, Zonguldak 67100, Türkiye; 3 Advanced Technologies Application and Research Center, Ankara Yıldırım Beyazıt University, Ankara 06031, Türkiye; 4 Laboratory of Useful Materials, National Institute of Research and Physicochemical Analysis, Technopole Sidi Thabet, Ariana 2020, Tunisia

**Keywords:** Zn-Ag-Fe alloy, nanocrystalline, biodegradable metal

## Abstract

Novel pure Zn and Zn-3Ag-xFe (x = 0, 1, 3, 5) (wt.%) nanocrystalline powders were synthesized for potential use as implants and stent materials by the mechanical alloying (MA) technique. The morphological and structural alterations of the powders milled for 5, 10, and 20 h were examined. SEM research revealed that during MA, the original elemental powder particles were subjected to a cold-welding process, subsequently fracturing in a brittle manner. The EDX spectra of the powders milled for 20 h indicated a uniform distribution of components. Laser diffraction particle size examination proved that the Zn-3Ag-1Fe alloy had the smallest particle size at 58.8 µm. XRD examination indicates the existence of AgZn_3_ and Fe_3_Zn_10_ intermetallic phases. The crystallite size diminishes with prolonged milling time, decreasing from 130 nm to 30 nm. The porosity rose from 11.62% for pure Zn to 15.35% in the Zn-3Ag-5Fe alloy, suggesting that the incorporation of Ag and the higher Fe ratio diminished the compressibility of the milled powders, as evidenced by density tests. The Zn-3Ag-5Fe alloy exhibited the maximum corrosion current density of 164.65 µA/cm^2^, attributed to the microgalvanic effect and reduced relative density induced by the Fe_3_Zn_10_ phase, which escalated with higher Fe doping. The hardness of the Zn-3Ag-5Fe alloy rose from 34.5 ± 2.8 HV to 132.2 ± 4.6 HV compared to the pure Zn sample, while the wear coefficient decreased from 0.029 ± 0.003 mm^3^/Nm to 0.005 ± 0.001 mm^3^/Nm, corresponding with the hardness test results. In contrast to *S. aureus*, which exhibited an 87.8% susceptibility to antibacterial activity from 3% silver and iron additions, *E. coli* demonstrated over 85% susceptibility to antibacterial activity from silver addition alone. The Zn-3Ag and Zn-3Ag-1Fe samples demonstrated high biocompatibility, attaining cell survival rates of 99.2% ± 3.01% and 99.2% ± 4.02% for the 12.5% extract, respectively. This study demonstrates that the newly developed Zn-Ag-xFe alloys have exceptional mechanical properties and excellent biocompatibility. Furthermore, the variable biodegradation rate dependent on alloy type presents an avenue for further research.

## 1. Introduction

Metallic implants are extensively utilized in orthopedic applications due to their superior mechanical characteristics. Bioinert implants have emerged as the predominant choice due to their superior corrosion resistance, fatigue, abrasion, and bending strength, together with their biocompatible nature [1].

Among bioinert metallic implants, stainless steels like 316L are identified by their cost-effectiveness and ease of machining. The outstanding corrosion and wear resistance of Co-Cr alloys, along with the exceptional biocompatibility of titanium alloys, are key factors causing to their extensive usage. The limited antibacterial and corrosion resistance of stainless steels, along with the high expense of Co-Cr alloys and the probable releasing of hazardous ions like nickel, cobalt, and chromium, provide limitations to their long-term application. The limited wear resistance and antimicrobial characteristics of titanium restrict its use. Additionally, the elastic modulus of all three bioinert implant types is significantly greater than that of cortical bone (20–30 GPa), which results in a stress-shielding effect. These unfavorable traits frequently call for a second surgical implant removal [2]. The most frequent cause reported by patients for removing implants was discomfort (59.5%). The aforementioned implant materials, which have a high elastic modulus, are the main source of discomfort. However, secondary implant surgeries are not advised unless absolutely required because of the risks of infection, nerve tissue damage, and incomplete healing after implant removal [3].

Recent years have seen a great deal of research into biodegradable implants, which aim to minimize the negative effects of conventional bioinert implants by gradually degrading inside the body and removing the need for secondary surgery. Among biodegradable implants, Mg, Fe, and Zn-based alloys are being intensively researched [4].

The most researched implant materials are magnesium alloys, which were the first biodegradable alloys to be developed. Their mechanical characteristics are quite similar to those of bone and offer exceptional biocompatibility. Moreover, they are appropriate for magnetic resonance imaging (MRI) applications due to their non-magnetic nature. However, magnesium degrades rapidly due to its highly reactive nature. The implant’s structural characteristics deteriorate quickly as a result of this degradation. Furthermore, magnesium decomposition causes the formation of H_2_ gas, which causes a rapid increase in pH around the tissues, leading to implant failure. Additionally, the utilization of iron-based biodegradable alloys is severely limited by their high elastic modulus, stress shielding effect, and slow rate of dissolution [5]. With an elastic modulus that is near bone (78–121 GPa) and a degradation rate that lies between that of magnesium and iron, zinc is a promising biodegradable metal [3]. Biologically, zinc is involved in about 300 enzyme activities that include cell proliferation, callus formation, and stimulation of the production of bone protein as it is involved in many aspects of cellular metabolism. Zinc is implicated in immune processes, the synthesis of protein and DNA, and the healing of wounds. The RDA and upper tolerance for zinc are 15 and 40 mg/day, respectively, and intake above these levels tends to be fairly non-toxic. Due to these biological processes, biodegradable zinc metal and its alloys have been the target of extensive research activity to increase mechanical, in vitro, and in vivo properties as bio-metallic implants [6]. Nevertheless, zinc alloys’ use in various plate, screw, orthopedic, and cardiovascular biomedical applications is restricted by mechanical features involving poor hardness, low wear resistance, and low bending and tensile strength [7]. A commonly used technique for enhancing the hardness, wear, and other mechanical characteristics of zinc alloys is binary and ternary alloying. When combined with elements like Mg, Li, Mn, Cu, Ca, Nd, and Sc, zinc exhibits exceptional mechanical qualities [8,9,10,11,12,13,14].

Zn-Ag alloys are also commonly employed in biodegradable zinc implant applications. Through processes including solid solution hardening, grain refining, and grain boundary precipitation, Ag not solely increases the hardness and tensile strength of zinc implants but also greatly boosts their antibacterial capabilities [15]. Zinc is alloyed with silver at different ratios of 0.4, 0.8, 2, 2.5, 4.5, 6, 7, and 8% and produced by traditional methods such as casting and hot extrusion or by new generation methods such as powder metallurgy and additive manufacturing [3,10,16,17]. In almost all studies, the incorporation of silver into zinc improved its mechanical properties and increased its corrosion rate. This indicates a widespread agreement in the literature about the mechanical characteristics and degrading parameters of Zn-Ag alloys. The consequences of incorporating a third element into Zn-Ag alloys, however, have not been extensively studied. Ledesma et al. [16] observed that the strength of a Zn-6Ag-0.5 Mg alloy rose from 72.7 to 371.5 MPa in comparison to pure Zn, whereas Mostaed et al. [18] claimed that adding 0.2–0.6% Mn to a Zn-4Ag alloy enhanced the mechanical characteristics. Iron is not only an essential element in the human body but also a biodegradable metal. The impact of adding iron, a high-strength biodegradable metal, as a third element to Zn-Ag alloys, has not yet been studied.

However, various techniques, such as casting [19], spark plasma sintering [20], cold pressing and sintering [21], forging [22] and extrusion [23], have been employed in the literature to fabricate Zn-Fe and Zn-Fe-x alloys. Numerous investigations have yielded conflicting results about the incorporation of iron into Zn-Fe and Zn-Fe-x alloys. In their electrochemical investigation of Zn, Zn-0.4Fe, and Zn-2.5Fe alloys produced through casting and subsequent extrusion, Su et al. [24] determined that the incorporation of 0.4% Fe enhanced corrosion resistance as evidenced by potentiodynamic polarization tests. The alloy, despite a minor decrease in corrosion resistance due to the incorporation of 2.5% Fe, nonetheless demonstrated improved corrosion resistance relative to pure zinc. Comparable behavior was seen by Xue et al. [19] and Zhang et al. [23]. In contrast to the aforementioned experiments, Kralova et al. [21] demonstrated that the incorporation of even 1% Fe into pure Zn elevated the corrosion rate by over 13-fold. Avior et al. also indicated that the incorporation of Fe elevated the corrosion rate [25]. The inconsistency in the biodegradation rate of Zn-Fe alloys is also evident in their biocompatibility and mechanical qualities. Although the incorporation of Fe typically enhances the hardness and tensile strength of these alloys, definitive findings concerning biocompatibility remain unreported [21,24,26,27]. Consequently, the impact of incorporating iron into zinc and its alloys necessitates validation through extensive research to determine the suitability of iron as an alloying element for zinc-based biomaterials.

Despite these advances, critical research gaps remain that limit the development of optimized biodegradable Zn alloys. First, while Zn-Ag binary alloys have demonstrated promising mechanical properties and antibacterial activity, the consequences of incorporating a third alloying element to modulate degradation rates and enhance mechanical performance have not been extensively investigated. Second, the literature on Zn-Fe alloys presents highly inconsistent findings regarding corrosion behavior, with reported corrosion rates varying by orders of magnitude depending on processing methods and Fe content, and conflicting evidence regarding biocompatibility. Third, and most importantly, the potential synergistic effects of combining Ag and Fe as dual alloying elements in a Zn matrix remain completely unexplored. The combined system could potentially harness Ag’s antibacterial properties and solid solution strengthening alongside Fe’s ability to form intermetallic phases that modulate degradation rates, but no prior studies have investigated this ternary Zn-Ag-Fe system.

Furthermore, the high melting point differences between zinc (419.5 °C), silver (961.8 °C), and iron (1538 °C) present substantial challenges for conventional casting-based fabrication methods due to zinc’s low melting and boiling points [28]. Powder metallurgy via mechanical alloying offers a unique solution by enabling the synthesis of alloys from metals with disparate melting points without requiring melting. However, powder metallurgy studies on zinc-based biodegradable alloys remain quite limited in the literature [2,28,29,30,31].

To address these critical gaps, this study aims to develop and characterize nanocrystalline Zn-3Ag-xFe (x = 0, 1, 3, 5 wt.%) alloys prepared by mechanical alloying for biomedical implant applications. This work represents the first investigation of the Zn-Ag-Fe ternary system, examining how Fe additions modulate the microstructure, mechanical properties, electrochemical degradation behavior, and biological performance of Zn-3Ag alloys.

## 2. Materials and Methods

### 2.1. Alloy Preparation and Consolidation

High-purity elemental powders of zinc (Zn, ≥99.9% purity, particle size < 44 µm; Nanokar, Istanbul, Türkiye), silver (Ag, ≥99.99% purity, particle size < 20 nm; Molchem, London, UK), and iron (Fe, ≥99.9% purity, particle size < 60 µm; Sigma-Aldrich, St. Louis, MO, USA) were used as starting materials. The alloy compositions were formulated as pure Zn and Zn-3Ag-xFe (where x = 0, 1, 3, and 5 wt.%). All powder handling and weighing were conducted in an argon-filled glove box (VGB-1, MTI Corporation, Richmond, CA, USA) to minimize oxidation.

Nanocrystalline alloy powders were produced by mechanical alloying (MA) using a high-energy planetary ball mill (PM-400; Retsch GmbH, Haan, Germany). For each composition, 20 g of the powder mixture was loaded into a hardened steel vial with hardened steel balls, maintaining a ball-to-powder weight ratio of 10:1. To minimize excessive cold welding, 1 wt.% stearic acid (C_18_H_36_O_2_) was added as a process control agent. The vials were sealed under argon atmosphere and milled at 200 rpm for 20 h. Powder samples were extracted at 5 and 10 h intervals for intermediate characterization.

The 20 h milled powders were uniaxially cold pressed into cylindrical pellets (13 mm diameter) at 750 MPa for 5 min using a hydraulic press. The green compacts were sintered in a tube furnace (Protherm, Istanbul, Türkiye) at 280 °C for 1 h under flowing high-purity argon to improve densification while preserving the nanocrystalline structure.

### 2.2. Microstructural and Phase Characterization

Phase identification was performed via X-ray diffraction (XRD; Empyrean, Malvern Panalytical, Worcestershire, UK) using Cu-K_α_ radiation (λ = 1.5406 Å) at 45 kV and 40 mA. Scans were conducted over a 2*θ* range of 10–90° with a step size of 0.013°.

Powder morphology, particle size evolution, and elemental distribution were examined using scanning electron microscopy (SEM; Quanta 450 FEG, FEI, Hillsboro, OR, USA) equipped with energy-dispersive X-ray spectroscopy (EDX). Particle size distribution was determined by laser diffraction analysis (Mastersizer 3000; Malvern Panalytical, Worcestershire, UK) with deionized water as the dispersant.

For sintered pellets, microstructural analysis involved standard metallographic preparation, grinding with SiC papers up to 2000 grit and polishing with a 6 µm diamond suspension.

### 2.3. Physical and Mechanical Properties

While evaluating the physical properties of the zinc-based samples, the basic characteristics of three consolidated samples for each alloy were evaluated before and after sintering. First, theoretical densities (*ρ_th_*) were calculated from the rule of mixtures presented in Equation (1) [32].

(1) $$\rho_{th}=\frac{m_{a}+m_{b}+m_{c}}{V_{a}+V_{b}+V_{c}}$$

Herein, *ρ_th_* is theoretical density (g/cm^3^); *m_a_*, *m_b_*, and *m_c_* show the weights (g) of the elements; and *V_a_*, *V_b_*, and *V_c_* show the volume (cm^3^) of these elements.

In addition to the theoretical density calculations, the actual density values of all samples were calculated according to Equation (2) using the Archimedes principle [33].

(2) $$\rho =\frac{M_{d}}{M_{d}-M_{s}}$$

Here, *M_d_* is the dry weight and *M_s_* is the suspended weight.

Additionally, the porosity ratios of the sintered consolidated samples were calculated according to Equation (3) [34].

(3) $$P=\left(1-\frac{\rho_{sample}}{\rho_{th}}\right)\times 100$$

Vickers microhardness (HV) measurements were performed on the polished surfaces of the sintered pellets according to the ISO 6507 standard [35], using a microhardness tester (HMV-G; Shimadzu Corp., Kyoto, Japan) with a 0.24 N load and 10 s dwell time. At least ten indentations per sample were averaged for statistical reliability.

Tribological behavior was evaluated using a ball-on-disk tribometer (Tribo Technic, Clichy, France) in a linear reciprocating mode, following the ASTM G133-05. Tests employed a 100Cr6 steel ball (6 mm diameter-58 HRC hardness) as the counterface under a 2 N normal load and 50 m sliding distance. The specific wear rate (mm^3^/N·m) was calculated from wear track volume measured by profilometry (Taylor Hobson, Leicester, UK) using the Archard equation [6].

### 2.4. Electrochemical Tests

The electrochemical corrosion behavior of the sintered pellets was evaluated in Hank’s Balanced Salt Solution (HBSS) at 37 ± 1 °C. The composition of the HBSS was (in mM): 138 NaCl, 5.33 KCl, 0.3 Na_2_HPO_4_, 0.44 KH_2_PO_4_, 1.3 CaCl_2_, 0.41 MgSO_4_·7H_2_O, 4 NaHCO_3_, 5.6 C_6_H_12_O_6_, and 0.5 MgCl_2_·6H_2_O.

A three-electrode cell was used with a potentiostat (Gamry Interface 1010, Gamry Instruments, Warminster, PA, USA). The sintered sample served as the working electrode, a saturated calomel electrode (SCE) as the reference electrode, and a graphite rod as the counter electrode. After immersing 30 min to stabilize open circuit potential (OCP), electrochemical impedance spectroscopy (EIS) tests were performed at OCP from 0.1 Hz to 10^5^ Hz with amplitude ±10 mV. Afterwards, potentiodynamic polarization curves were recorded by scanning the potential from −500 to +500 mV relative to the OCP at a scan rate of 0.5 mV/s. Corrosion potential (E__corr_) and current density (i__corr_) were determined by Tafel extrapolation of the polarization curves, and corrosion rate was calculated based on ASTM G59-97 [36,37].

### 2.5. In Vitro Biocompatibility Assessment

#### 2.5.1. Cytotoxicity Assay Procedure

The in vitro cytotoxicity of the alloys was evaluated according to ISO 10993-5 using an indirect extract method. Mouse osteoblast precursor cells (MC3T3-E1, ATCC CRL-2593) were cultured in alpha-Minimum Essential Medium (α-MEM) supplemented with 10% Fetal Bovine Serum (FBS) and 1% penicillin-streptomycin at 37 °C in a 5% CO_2_ humidified atmosphere.

Alloy extracts are used to determine the toxicity of alloys. The extract was prepared by incubating the alloy (0.2 g/mL *w*/*v*) in α-MEM cell culture medium and incubating at 37 °C for 72 h (ISO 10993-12). Extracts were used at 1× (undiluted) and 5× dilutions. MC3T3-E1 cells were seeded in 96-well plates (1 × 10^4^ cells/well) and incubated for 24 h. The medium was replaced with the prepared extracts (1× and 5×) and the cells were incubated for another 24 h 37 °C in a 5% CO_2_ incubator. Cell viability was quantified using a Cell Counting Kit-8 (CCK-8) assay (Dojindo, Kumamoto, Japan). The optical density (OD) was measured at 450 nm using a microplate reader (CLARIOstar Plus, Ortenberg, Germany). Cells cultured in fresh medium served as the control group (100% viability).

#### 2.5.2. Live/Dead Staining

Cell viability and morphology of cells exposed to the alloy extracts were visualized using a live/dead staining kit. In this assay, live cells, due to their intact cell structure and intracellular enzymatic activity, take up Calcein AM and appear green. Dead cells, due to plasma membrane damage, appear red due to nuclei stained with 7-ADD.

MC3T3-E1 cells (2 × 10^4^ cells/slide) were cultured on slides to ensure adherence and were exposed to 1× and 5× alloy extracts for 24 h. For fixation, the cells were washed three times with PBS and incubated with 4% formalin for 30 min at room temperature. Calcein AM and 7-ADD dyes were added to the fixed cells and incubated at 37 °C for 15–20 min. Following incubation, cells were washed with phosphate-buffer solution (PBS), covered with a coverslip, and visualized with a fluorescence microscope (Carl Zeiss, Munich, Germany). For positive control, cells were incubated with medium and for negative control, with ethanol for 30 s.

### 2.6. Antibacterial Activity Test

The antibacterial properties of the alloys were assessed against Gram-positive *Staphylococcus aureus* (ATCC 29213) and Gram-negative *Escherichia coli* (ATCC 25922) using a direct contact colony-forming unit (CFU) counting method. Bacterial suspensions were prepared in nutrient broth and standardized to a 0.5 McFarland turbidity (~1.5 × 10^8^ CFU/mL). Sterile samples were placed in 2 mL of the bacterial suspension and incubated for 24 h at 37 °C.

After incubation, samples were washed with PBS to remove loosely attached bacteria. Adherent bacteria were then detached by sonication in fresh PBS. The resulting suspensions were serially diluted, plated onto nutrient agar plates, and incubated for 24 h at 37 °C. The number of CFUs was counted to determine the antibacterial efficacy of each sample.

In addition, bacteria attached to the surface of the alloy were visualized by SEM. For this, the samples were incubated in 5 mL of bacterial suspension (McFarland 0.5) at 37 °C for 24 h. Bacteria adhered to the samples were fixed with 4% paraformaldehyde and then dehydrated with ethanol (30, 50, 70, 80, 90, 95, and 100%) gradient dehydration to remove water from the samples.

### 2.7. Statistical Analysis

All quantitative data are presented as mean ± standard deviation (*n* = 3, unless otherwise stated). Statistical significance was determined using a one-way analysis of variance (ANOVA) followed by Tukey’s post hoc test for multiple comparisons. A *p*-value of <0.05 was considered statistically significant.

## 3. Results and Discussion

### 3.1. Structural and Phase Evolution During Mechanical Alloying

The structural changes in pure Zn and Zn-3Ag-xFe (x = 0, 1, 3, and 5 wt.%) powders during 5, 10, and 20 h of milling were investigated using XRD. The XRD patterns of pure Zn powder milled for various durations are presented in Figure S1a. For the as-received powder (0 h), sharp diffraction peaks corresponding to the hexagonal close-packed (hcp) structure of Zn (ICSD ref. no: 98-018-1734) are clearly visible. With increasing milling time, a significant broadening and a decrease in the intensity of these characteristic peaks were observed. This is a well-established consequence of severe plastic deformation induced by MA, indicating substantial crystallite size refinement and an accumulation of internal lattice strain [38]. The crystallite size and lattice strain were calculated from the full width at half maximum (FWHM) of the most intense Zn peak at 2*θ* ≈ 43° using the Debye-Scherrer and Wilson-Stoke’s equations, respectively [39]. As shown in Figure S1b, the crystallite size of pure Zn decreased precipitously to approximately 57 nm within the first 5 h of MA, eventually reaching a near-steady state value of ~43 nm after 20 h of MA. Correspondingly, the lattice strain progressively increased, reaching approximately 0.23% after 20 h of MA, which is attributed to the high density of crystal defects, such as dislocations, generated during the continuous fracture and cold-welding events inherent to the MA process [40].

For the binary Zn-3Ag alloy, the XRD patterns of un-milled powders (Figure S2a) show distinct peaks for both hcp-Zn (ICSD ref. no: 98-024-7159) and face-centered cubic (fcc) Ag (ICSD ref. no: 01-087-0717), as expected. After 5 h of MA, the elemental Ag peaks are no longer detectable, and new peaks corresponding to the AgZn_3_ intermetallic phase (ICSD ref. no: 00-025-1325) emerge. Concurrently, the intensity of the Zn peaks diminishes. As milling progresses to 20 h, the dominant phases remain hcp-Zn and AgZn_3_. It is noteworthy that we successfully synthesized the AgZn_3_ phase in a Zn-3Ag composition via MA. This is in contrast to reports on cast alloys, where Liu et al. [41] did not observe this phase in alloys with less than 3.5 wt.% Ag, and Sikora-Jasinska et al. [42] reported its formation only at 5 and 7 wt.% Ag. This highlights the non-equilibrium processing capability of MA to facilitate intermetallic phase formation at lower solute concentrations. As seen from Figure S2b, the crystallite size drops from over 130 nm to 75 nm in the first 5 h and is further refined to a final size of ~35 nm after 20 h of MA. The accumulated lattice strain after 20 h was calculated to be ~0.28%, higher than that of pure Zn, likely due to the additional strain introduced by the formation of the AgZn_3_ intermetallic phase.

For the ternary Zn-3Ag-xFe (x = 1, 3, and 5) alloys, the XRD patterns for the un-milled powders displayed overlapping peaks from elemental Zn, Ag, and Fe (ICSD ref. no: 98-018-5748), as seen in Figure S3. After 5 h of MA, the elemental Ag and Fe peaks vanished across all compositions. Concurrently, peaks corresponding to the AgZn_3_ and Fe_3_Zn_10_ (ICSD ref. no: 01-071-0399) intermetallic phases were identified. These phases remained stable throughout the 20 h of MA, indicating that the final phase constitution was achieved relatively early.

**Figure 1:**
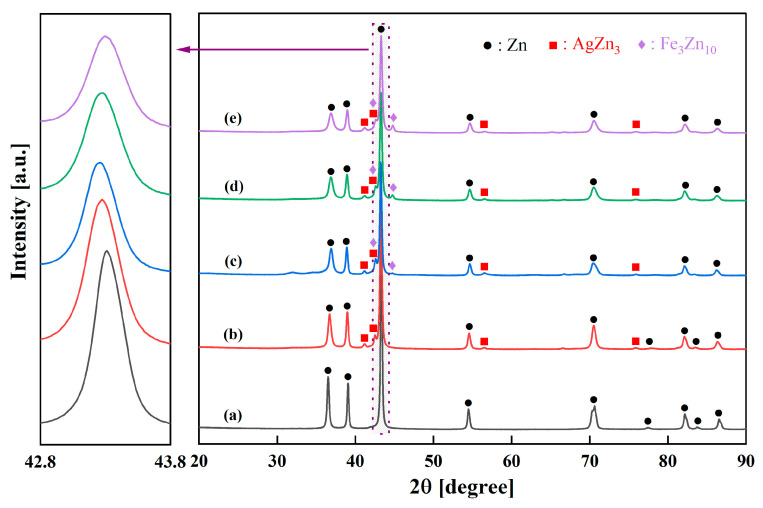
XRD patterns of 20 h milled powder alloys: (**a**) pure Zn, (**b**) Zn-3Ag, (**c**) Zn-3Ag-1Fe, (**d**) Zn-3Ag-3Fe, and (**e**) Zn-3Ag-5Fe.

Figure 1 presents a comparative overview of the XRD patterns for all compositions after 20 h of MA. A subtle but significant shift in the position of the main Zn (101) peak in the range of 2*θ* ≈ 42.8–43.8° is evident. In the Zn-3Ag alloy (Figure 1b), this peak shifts to a lower 2*θ* angle compared to pure Zn (Figure 1a). This is consistent with an expansion of the Zn lattice due to the substitution of smaller Zn atoms (empirical atomic radius of 135 pm) with larger Ag atoms (atomic radius of 160 pm). Conversely, with the addition of Fe (atomic radius of 140 pm), the peak shifts back towards higher 2*θ* angles (Figure 1c–e). This indicates a complex change in the lattice parameters resulting from the co-dissolution of Ag and Fe into the Zn matrix and the precipitation of the intermetallic phases.

The evolution of crystallite size and lattice strain for the ternary Zn-Ag-Fe alloys is presented in Figure S4. The Zn-3Ag-1Fe alloy (Figure S4a) exhibited rapid initial refinement, reaching a crystallite size of ~36.5 nm after 5 h and a final size of ~34 nm at 20 h. In contrast, the Zn-3Ag-3Fe and Zn-3Ag-5Fe alloys (Figure S4b,c) showed a more gradual refinement, decreasing from ~73 nm at 5 h to ~30 nm at 20 h. The lattice strain increased with milling time for all ternary alloys, reaching a maximum of ~0.28% for Zn-3Ag-1Fe and ~0.32% for the alloys with higher Fe content. This increased strain is a direct result of the severe plastic deformation induced by the milling media and the lattice distortion from the alloying elements [38].

**Figure 2:**
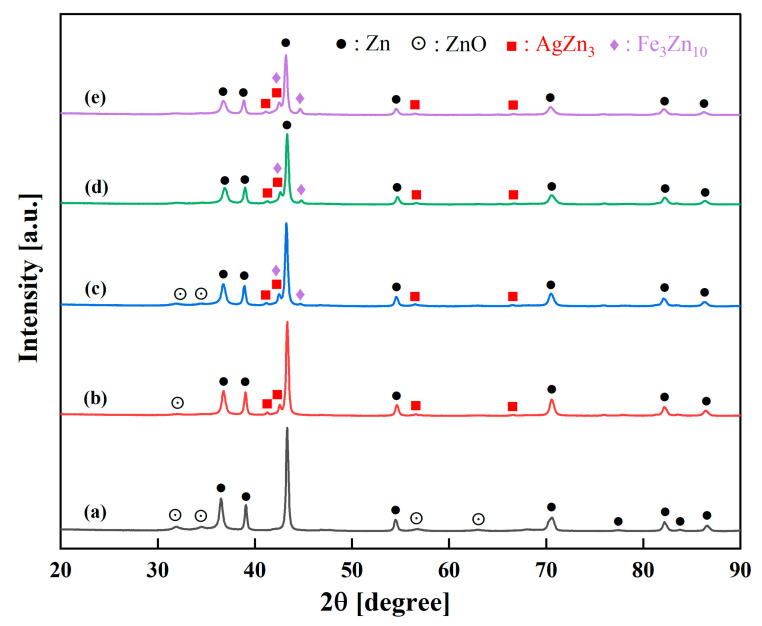
XRD patterns of the consolidated samples: (**a**) pure Zn, (**b**) Zn-3Ag, (**c**) Zn-3Ag-1Fe, (**d**) Zn-3Ag-3Fe, and (**e**) Zn-3Ag-5Fe.

The XRD patterns of the consolidated samples produced by cold pressing and sintering the milled powders for 20 h are presented in Figure 2. All consolidated samples exhibit the characteristic diffraction peaks of the hcp zinc phase, which remains the dominant constituent in all compositions. For the consolidated pure Zn sample (Figure 2a), the XRD pattern displays well-defined peaks corresponding to the hcp-Zn phase along with minor ZnO peaks. The presence of zinc oxide can be attributed to surface oxidation during powder handling and processing, consistent with previous observations in mechanically processed zinc-based materials where exposure to atmospheric conditions leads to superficial oxide layer formation. The binary Zn-3Ag alloy (Figure 2b) shows the emergence of distinct peaks corresponding to the AgZn_3_ phase. This finding aligns with established Zn-Ag phase diagrams where AgZn_3_ formation occurs at Ag concentrations above approximately 2.5–3 wt.% [41,42,43]. Similarly to the as-milled powders in Figure 1, for the ternary Zn-3Ag-xFe alloys (Figure 2c–e), peaks corresponding to both the AgZn_3_ and Fe_3_Zn_10_ intermetallic phases are present alongside the Zn phase. The relative intensity of the Fe_3_Zn_10_ peaks at around 2*θ* = 44.7° systematically increases with higher Fe content, which is consistent with the nominal compositions of the alloys.

### 3.2. Morphological Evolution During Mechanical Alloying

The morphological evolution of the pure Zn and Zn-3Ag-xFe (x = 0, 1, 3, and 5 wt.%) alloy powders during MA was investigated using SEM-EDX. Initial pure Zn powders exhibited elongated, needle-like morphology (Figure S5). During early milling stages, particles underwent cold welding, forming large layered agglomerates. As milling progressed, these agglomerates underwent significant work hardening, increasing their brittleness. Consequently, the primary mechanism shifted to fracturing, which broke down the large agglomerates. After 20 h of milling, a steady-state equilibrium between cold welding and fracturing was established, resulting in a more uniform distribution of smaller, nearly equiaxed powder particles. A similar evolutionary pattern was observed for the alloyed powders, as shown for Zn-3Ag, Zn-3Ag-1Fe, Zn-3Ag-3Fe, and Zn-3Ag-5Fe in Figures S6, S7, S8, and S9, respectively. The addition of ductile Ag appeared to enhance the initial cold-welding effect. For all compositions, continued milling up to 20 h refined the particle size and improved morphological homogeneity. This proves that MA significantly impacts both particle size and homogeneity of the powders.

The particle size distribution of the powders after 20 h of milling was evaluated using laser diffraction as shown in Figure S10. The median particle size (Dv50) was found to be dependent on the alloy composition. The finest powder was the Zn-3Ag-1Fe alloy with a Dv(50) value of 58.8 µm. In comparison, pure Zn and the binary Zn-3Ag alloy showed slightly larger median particle sizes of 62.5 and 64.6 µm, respectively. However, increasing the Fe content led to a coarser final powder, as the Zn-3Ag-3Fe and Zn-3Ag-5Fe alloys exhibiting the largest Dv(50) values of 86.0 and 99.1 µm, respectively. This suggests that higher concentrations of Fe alter the dynamic equilibrium between the cold welding and fracturing mechanisms, promoting the formation of larger agglomerates at the steady state.

**Figure 3:**
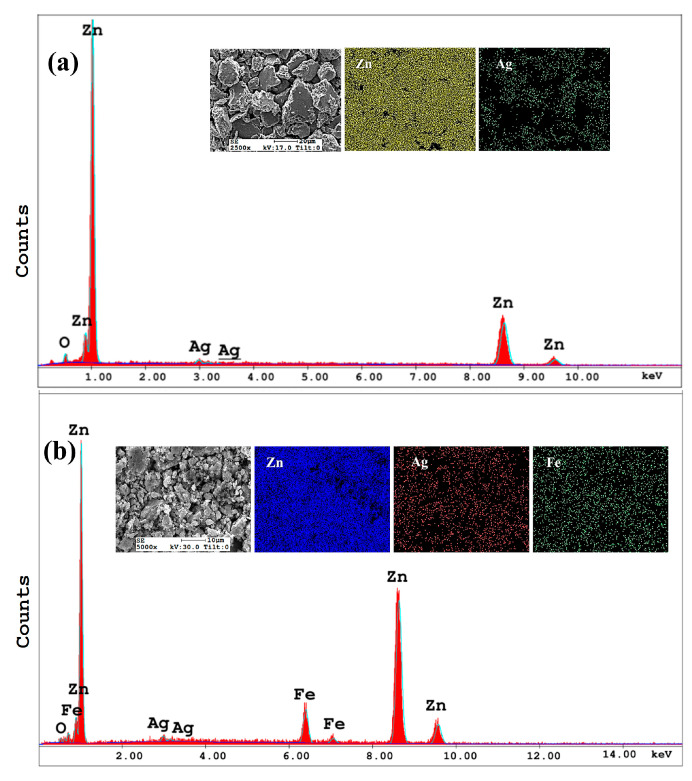
SEM-EDX spectra and elemental mapping images of 20 h milled powder alloys: (**a**) Zn-3Ag and (**b**) Zn-3Ag-5Fe.

As seen in Figure 3, the EDX analysis of powders after 20 h of MA confirmed successful elemental homogenization. Elemental mapping revealed uniform distribution of Zn, Ag, and Fe throughout the powder particles in both Zn-3Ag and Zn-3Ag-5Fe alloys. This homogeneous distribution validates the effectiveness of the 20 h processing duration. However, EDX spectra showed trace oxygen content, attributed to minor surface oxidation during powder handling and processing despite the taken precautions.

**Figure 4:**
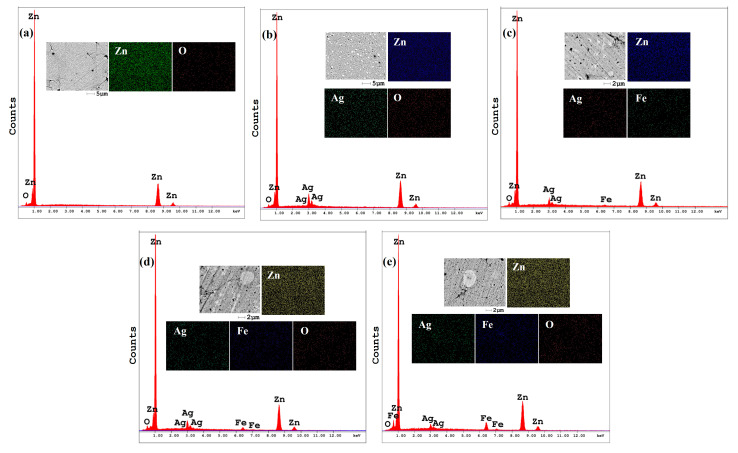
SEM-EDX spectra and elemental mapping images of the consolidated samples: (**a**) pure Zn, (**b**) Zn-3Ag, (**c**) Zn-3Ag-1Fe, (**d**) Zn-3Ag-3Fe, and (**e**) Zn-3Ag-5Fe.

The SEM-EDX spectra and elemental mapping images of the consolidated pure Zn and Zn-3Ag-xFe (x = 0, 1, 3, and 5 wt.%) samples are presented in Figure 4. The consolidated pure Zn sample (Figure 4a) exhibits a relatively uniform microstructure dominated by Zn, with trace oxygen detection attributed to surface oxidation during powder handling and processing. The minor ZnO phase was detected in the XRD pattern of the sample (Figure 2a). For the Zn-3Ag alloy (Figure 4b), the microstructure is characterized by a fine, uniform dispersion of a bright secondary phase within the Zn matrix. The elemental map for Ag confirms that this phase is Ag-rich, corresponding to the AgZn_3_ intermetallic identified by XRD (Figure 2b). For the ternary Zn-3Ag-xFe alloys (Figure 4c–e), the EDX elemental mapping confirms uniform distribution of Zn, Ag, and Fe elements. In addition to the Zn matrix and the bright Ag-rich phase (AgZn_3_), a third phase with an intermediate gray contrast is visible. Elemental mapping for Fe confirms that this phase is Fe-rich, corresponding to the Fe_3_Zn_10_ intermetallic also identified by XRD (Figure 2c–e).

### 3.3. Density and Mechanical Properties

The consolidated Zn and Zn-3Ag-xFe (x = 0, 1, 3, and 5 wt.%) samples before and after sintering are shown in Table 1. According to table, the compressibility of pure zinc powders had the highest value both before and after sintering. The sintering process increased the void ratio by 6% or more for each sample. With the addition of 3% Ag to the Zn element, solid solution zinc and AgZn_3_ intermetallic compounds are formed, according to the XRD pattern in Figure 2, the SEM/EDX images in Figure 4, and the previous studies [14,44,45]. Intermetallic phases have a harder structure than the matrix phase, and in zinc production by powder metallurgy, hard phases and additions reduce compressibility in production [46,47]. Intermetallic compounds stand out among alloys with their brittle structures, lower ductility and higher hardness properties. Yan et al. [48] also showed that increasing intermetallic compounds with increasing Zn content increased the porosity rate of Mg-based biodegradable Mg-Zn alloys. Similarly, the Fe_3_Zn_10_ intermetallic compound formed by the addition of iron reduced the cohesiveness of the alloy powders, causing higher porosity [21].

**Figure 5:**
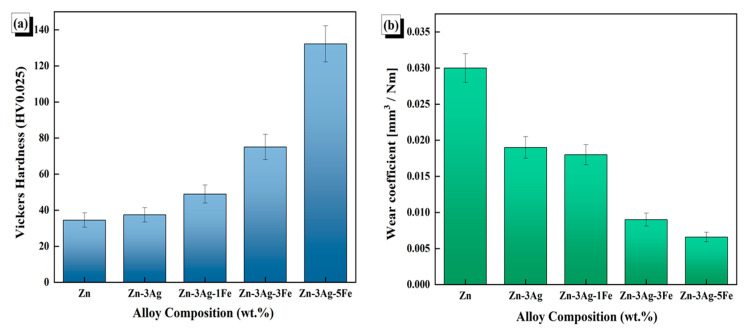
Effect of Fe content on the (**a**) Vickers microhardness and (**b**) wear behavior of the consolidated Zn-3Ag alloys.

The microhardness values are displayed in Figure 5a, whereas the microhardness photographs of the sintered samples are displayed in Figure S11. For every sample, microhardness tests were conducted three times. The Zn-3Ag-3Fe and Zn-3Ag-5Fe samples had a greater percentage of AgZn_3_ and Fe_3_Zn_10_ intermetallics and a higher hardness in tests conducted at 400× magnification using Shimadzu HMV-G equipment. The standard deviation in the hardness tests rose as the percentage of intermetallic compounds increased. Through solid solution hardening [45] and precipitation hardening, with the help of intermetallic precipitates [49], Ag addition enhances the mechanical characteristics of zinc-based biodegradable metals. As seen in the SEM/EDX images of the Zn-3Ag and Zn-3Ag-1Fe samples, the intermetallic phases are finely and homogeneously distributed. Similarly, Ag is 2.6% soluble in Zn at room temperature. However, SEM/EDX images show proportionally higher levels in all alloys. This suggests that silver is both dissolved in the zinc and present as a secondary phase [50]. Accordingly, increasing the secondary phase ratio with the addition of iron increased the hardness to 132.8 HV, resulting in an alloy much harder than many zinc alloys and composites reported in the literature. Figure 5b shows the wear coefficient values calculated using the Archard’s equation. According to this equation, the decrease in the wear coefficient indicates a decrease in the wear rate of the samples. Wear resistance, which is often directly proportional to an increase in hardness [51], has been studied only limitedly for biodegradable zinc alloys [52,53]. The Zn-3Ag-5Fe alloy, which achieves significantly higher hardness with an increase in the intermetallic phase ratio, has been identified as the most suitable alloy for applications requiring wear resistance.

### 3.4. Electrochemical Properties

**Figure 6:**
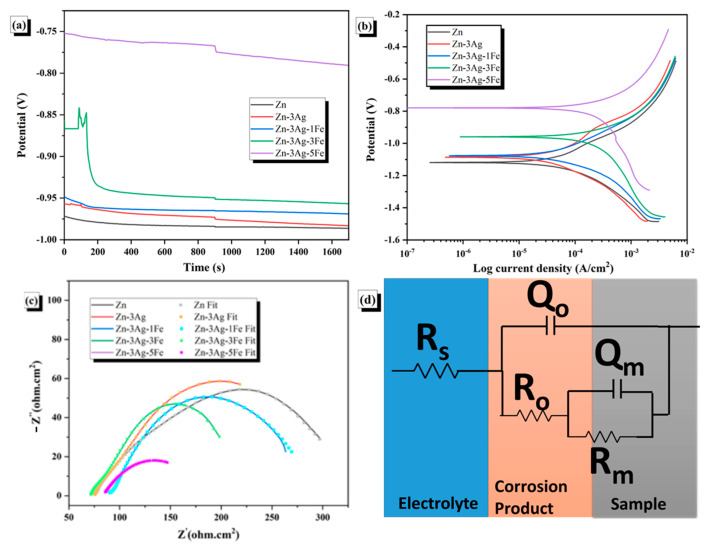
Electrochemical test results. (**a**) Open Circuit Potential (OCP), (**b**) potentiodynamic polarization, (**c**) EIS and (**d**) equivalent circuit.

Figure 6 illustrates the results of electrochemical tests. The open circuit potential increases with the addition of Ag and then iron, as can be observed by considering the open circuit potential values in Figure 6a. Ag (+0.8 V) and Fe (−0.440 V) have standard electrode potentials, but Zn has a standard electrode potential of −0.76 V [54,55]. This large difference caused a significant change in open circuit potential (OCP) and corrosion potential. A similar trend was also observed by Yılmazer et al. [45]. The corrosion potential of pure zinc metal may be negatively impacted when silver is added. Sikora et al. [42], for instance, found that adding Ag to cast zinc reduced the pure zinc’s corrosion potential from −0.98 to −1.12 V, resulting in more corrosive behavior. On a contrary, Shuai et al. [17] showed that the addition of Ag raised the corrosion potential of Zn and Zn-xAg alloys made by selective laser melting. Despite this contradiction in the literature regarding the effect of Ag addition on corrosion potential and open circuit potential values, it has been observed in almost all studies that alloying accelerates corrosion by creating microgalvanic effects with the matrix by forming phases such as AgZn_3_ [14]. In our work, the corrosion current density of pure Zn rose from 29.44 µA/cm^2^ to 33.12 µA/cm^2^ with the incorporation of 3% Ag. The AgZn_3_ phase elevated the electrochemical corrosion rate as anticipated.

The impact of iron incorporation into zinc-based alloys is notably inconsistent in the literature. The corrosion current densities of Zn-2Fe alloys were measured at 37.17 µA/cm^2^ [20], 0.82 µA/cm^2^ [56] and 2.22 µA/cm^2^ [57]. The identical Zn-Fe alloys demonstrate varying corrosion rates, and the influence of iron on zinc corrosion rates remains highly contentious. According to Guan et al., the corrosion rate of pure zinc escalated during the immersion test due to the development of the FeZn_13_ intermetallic phase upon the addition of 2% iron, whereas the corrosion current density remained unchanged in the electrochemical corrosion test of the identical sample [56]. Su et al. [24] demonstrated that the electrochemical corrosion rate of the Zn-0.4 Fe and Zn-2.5 Fe alloys decreased with increasing iron content. The decrease in corrosion rate was attributed to the passive film formed by the thin, homogeneous FeZn_15_ phase. In a similar vein, Zhang et al. [23] observed that the addition of iron to zinc led to the formation of the FeZn_13_ phase, which subsequently decreased the corrosion rate by generating a passive film. Li et al. [20] found that incorporating iron into zinc produces the FeZn_13_ phase, which subsequently induces microgalvanic effects due to the potential difference between the zinc and the Zn matrix. Consequently, augmenting the iron concentration (1%, 1.5%, and 2%) elevates the intermetallic phase ratio, thereby significantly accelerating the corrosion rate. Kafri et al. demonstrated that the in vivo corrosion of Zn and Zn-2Fe alloys implanted in rats revealed a more than twofold increase in corrosion rate with the addition of 2% iron [26]. In our study, unlike previous studies, neither the FeZn_13_ nor FeZn_15_ phases were observed. The XRD results indicate the presence of the Fe_3_Zn_10_ intermetallic phase in the structure, independent of the iron addition. The proportion of this phase increased with higher iron concentrations. The mechanical alloying method facilitates the contact between two elements due to the high energy requirements involved. Application of sufficiently high energy typically enhances mutual solubility in mechanical alloying. The increased solubility and higher energy facilitate the formation of intermetallic phases that are absent in standard phase diagrams [58]. SEM/EDX images and XRD data showed that as Fe ratio increased so did the percentage of the intermetallic phase. Considering the matrix as an anode and the intermetallic phase as a cathode, a microgalvanic effect occurs between the matrix and intermetallic phase. The higher the cathodic phase, the faster the corrosion rates occur [21]. The iron concentration in the Fe_3_Zn_10_ phase significantly exceeds that of FeZn_13_ and FeZn_15_. This may have resulted in a more pronounced disparity in the electrode potential of the Fe_3_Zn_10_ phase relative to the zinc matrix, in contrast to the FeZn_13_ and FeZn_15_ phases. The significant potential difference may have led to an increased rate of microgalvanic reactions, resulting in the rapid degradation of the zinc matrix.

Figure 6b illustrates the Tafel curves for Zn, Zn-3Ag, and Zn-3Ag-xFe (x = 1, 3, and 5) alloys. Table 2 shows the potentiodynamic polarization test results, showing the highest corrosion rate of 2.81 mm/year in the Zn-3Ag-5Fe compound, while the lowest rate of 0.51 mm/year in the pure Zn sample.

As mentioned above, the corrosion rate depends not only on the intermetallic phase and matrix type but also significantly on the manufacturing method. Depending on the manufacturing method, the porosity in the structure also affects the corrosion rate. Increasing porosity increases the surface area in contact with the corrosive environment, which in turn accelerates corrosion. High porosity, which disrupts the continuity of the passive oxide film formed on the surface, accelerates the corrosion of parts manufactured using powder metallurgy [36,59]. Kralova et al. [21] observed that the formation of the Zn_13_Fe and Zn_11_Fe phases in the Zn-xFe (x = 0, 1, 2, 5, and 10) alloys, produced through powder metallurgical techniques, was enhanced with a rise in iron content. The investigation revealed that the Zn-10Fe alloy characterized by elevated porosity attributed to the increased iron content alongside the intermetallic phase, exhibited the highest corrosion current density. The corrosion current density of the Zn-10Fe alloy characterized by a porosity exceeding 35% was measured at 1203.3 ± 2.8 μA/cm^2^. In contrast, pure zinc which exhibited a porosity of 8.52% displayed a significantly lower corrosion current density of merely 7.34 ± 0.2 μA/cm^2^. The notable disparity can be attributed to the markedly enhanced porosity and the subsequent adsorption of the solution into the micropores created by this porosity, leading to accelerated degradation at the solution-metal interface. Alongside the influence of AgZn_3_ and Fe_3_Zn_10_ intermetallic phases as illustrated in the existing literature, the significant porosity present in the Zn-3Ag-3Fe and Zn-3Ag-5Fe alloys resulted in exceedingly rapid corrosion, akin to the findings of the aforementioned study.

Figure 6c shows the Nyquist curves for Zn, Zn-3Ag, and Zn-3Ag-xFe (x = 1, 3, and 5) alloys in 37 °C Hank’s Solution, and the equivalent circuit diagram drawn in Figure 6d based on these curves. Two distinct capacitive arcs are observed in the high-frequency region where the solution resistance is present and in the low-frequency region where the capacitance of the corrosion products is present. This indicates that the electrochemical behavior of samples in solution is controlled by two different components. The electrochemical behavior of zinc alloys is determined by the solution, the oxide film under the zinc substrate, and the behavior of the zinc alloy matrix. In the equivalent circuit constructed considering these two factors, *R_s_* represents the solution resistance, *R_o_* represents the corrosion product resistance on the zinc surface, and *R_m_* represents the charge transfer resistance of the zinc alloy matrix due to the oxidation of zinc ions. *Q_o_* represents the capacitance of the corrosion products, and *Q_m_* represents the capacitance between the zinc alloy and the solution [8]. According to the EIS results, the most important parameters for the corrosion resistance of the samples are the *R_o_* and *R_m_* components. This is because increased resistance to corrosion products and zinc ion diffusion indicates increased corrosion resistance. Accordingly, the total *R_o_* and *R_m_* resistance values, as seen in Table 3, are Zn > Zn3Ag > Zn3Ag1Fe > Zn3Ag3Fe > Zn3Ag5Fe. These results are consistent with the potentiodynamic polarization tests [60]. Since *R_o_* resistance is the film resistance formed on the surface as a result of corrosion, the continuity of the oxide film is disrupted with the increase in porosity, and the increase in porosity decreases the *R_o_* resistance.

### 3.5. Cytotoxicity Assay

For biodegradable bone implants, the formation of new bone tissue and the development of this tissue as the metal degrades play a critical role in healing. Therefore, the viability of the MC3T3-E1 mouse osteoblast cells used plays a critical role in Zn-based biodegradable bone implants [9]. According to the ISO 10993-5:2009 standard [61], when the cell viability rate falls below 70% compared to the control group, the sample is considered cytotoxic material [62].

**Figure 7:**
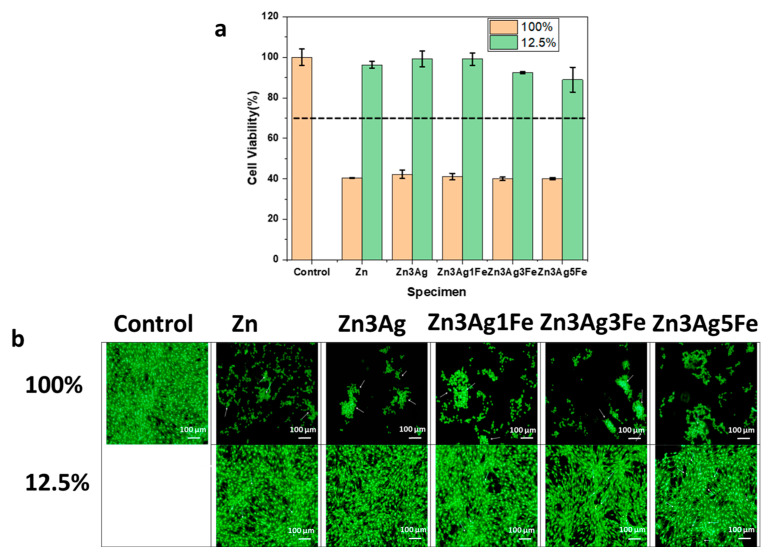
Cytocompatiblity of Zn, Zn3Ag, and Zn3AgxFe (x = 1, 3, and 5) alloys. (**a**) Cell viability of MC3T3-E1 Cells cultured in 100% and 12.5% extracts. *p* < 0.05. The dashed line indicates the toxic limit according to the ISO 10993-5 standard. (**b**) Live/dead staining of MC3T3-E1 cells. Green fluorescence indicates live cells, and white arrows indicate dead cells.

The cell viabilities of 100% and 12.5% extracts of pure zinc, Zn-3Ag, and Zn-3Ag-xFe (x = 1, 3, and 5) alloys are displayed in Figure 7a. According to the ISO 10993-5:2009 standard, all samples in 100% extract are toxic. Despite the fact that there is no discernible variation in the cell viabilities, the Zn-3Ag alloy had the greatest cell viability rate, at 42.19 ± 2.09%. The Zn3Ag3Fe and Zn3Ag5Fe alloys had the lowest viability, averaging 39.94 ± 0.83 and 40.087 ± 0.50%, respectively. When compared to the 100% extract, the cell viability rates in the 12.5% extracts showed a notable rise. Cell viability was above 85% for every sample. The greatest result, 99.20 ± 4.02%, is found in the Zn-3Ag alloy. While all other alloys show great biocompatibility with values above 90%, Zn3Ag5Fe has the lowest cell viability at 88.96%. In cytotoxicity tests on biodegradable metals, the metal ions corroded and oxidized primarily indicate the toxicity of the sample. The primary biodegradation ion in zinc-based metals is Zn^+2^. Therefore, the Zn^+2^ ratio significantly affects biocompatibility. In addition to the zinc ion, it is anticipated that under normal conditions, high silver ion degradation will exhibit toxic effects. However, researchers have been unable to demonstrate a clear cytotoxic effect of Ag on zinc alloys in many Zn-Ag studies because the decay rates of released Ag^+^ ions were below 50 µg/L [14,50,63].

Additionally, when evaluating the effects of Ag in the literature, cell tests were performed on different extracts. Because the values of released silver ions differ significantly from the lethal concentration (LC50) values, the direct toxicity effect of silver could not be fully determined. Furthermore, because there is no extraction standardization for biodegradable alloys, each study performs its tests on different extracts [14]. For instance, Shi et al. demonstrated that cell viability was decreased when Ag was added to the Zn0.8Mn alloy in 100% extract, but this was not the case in 80, 60, 40, and 20% extracts [62]. Xiao et al. also showed conflicting results for each extract and could not establish a clear correlation between Ag addition and cell viability [50]. Similarly to Ag, little is known about the impact of iron addition. However, it has been shown that cell viability is impacted by the ion release, which is contingent upon the rate of biodegradation in the Zn-0.5Fe alloy [22].

This is in line with the observation that limited cell viability was seen in both 100% and 12.5% extracts of the Zn-3Ag-5Fe alloy, which had the greatest corrosion rate in electrochemical testing. The effects of the substances on MC3T3-E1 cell viability are demonstrated by live/dead double-staining examination in Figure 7b [64]. This enables us to assess how the materials’ cytotoxicity affects cell integrity. The 7-ADD dye colors the cell nucleus red in dead cells, while the Calcein dye, which is reliant on enzyme activity (esterase), turns live cells green. The viability percentages from the CCK-8 study and the data from live-dead staining are in agreement. Images resembled those in the control group, and cells exposed to 12.5% material extract largely retained their cellular viability and integrity. On the surface, the cells were evenly spaced, and they were proliferating. However, cells cultured with 100% material extract showed a decrease in cellular viability. Cells clustered to stick to the slide surface as proliferation was decreased. Cell size reduction in comparison to the control group was used to measure cellular integrity. Furthermore, Figure S13 displays detailed pictures.

### 3.6. Antibacterial Activity

**Figure 8:**
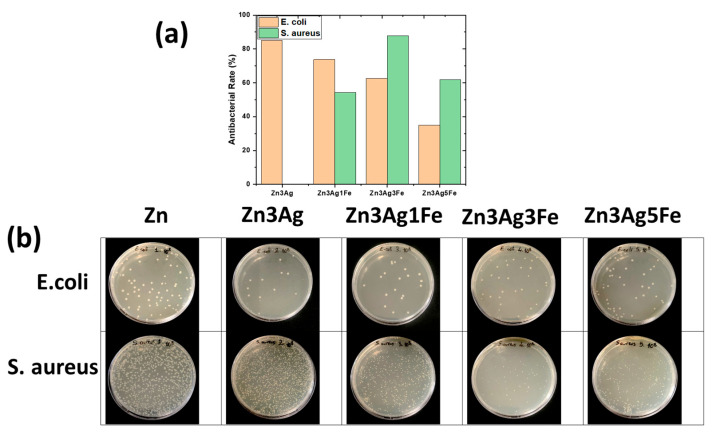
(**a**) Antibacterial rate of specimens relative to pure Zn. (**b**) *E. coli* and *S. aureus* bacterial colonies on Zn, Zn3Ag, and Zn3AgxFe (x = 1, 3, and 5 wt.%) alloys after incubation at 37 °C for 24 h.

Figure 8a shows the antibacterial rate, and 8b illustrates *E. coli* and *S. aureus* bacterial colonies. Zn, Zn-3Ag, and Zn-3Ag-xFe (x = 1, 3, and 5) alloys showed different activity against Gram-positive (*S. aureus*) and Gram-negative (*E. coli*) bacteria. In all samples, the antibacterial activity against Gram-negative bacteria was significantly greater than against Gram-positive bacteria. Increasing silver content indicated an increased antibacterial effect in zinc alloys [50]. The Zn-3Ag alloy had the strongest antibacterial action against *E. coli* with over 85% activity. Even in alloys with the same composition, adding silver and increasing corrosion rates can have distinct antimicrobial effects. In their experiments with *E. coli* bacteria in bulk and porous Zn-1Ag alloys, Xie et al. [44] discovered that the antibacterial activity of the porous structure was noticeably greater. Additionally, they showed that when 1% Ag was used to create the porous sample instead of 3.5%, the antibacterial activity nearly doubled. This demonstrates that corrosion rate is one of the key criteria for antibacterial activity. For *S. aureus*, in particular, increased corrosion rate, rather than Ag addition, has been shown to have a higher antibacterial effect. The effect of Fe addition to Zn-Ag alloys has not been studied in the literature. However, the effect of iron addition to zinc has been studied in a very limited number of studies. Su et al. demonstrated that increased iron had an increased antibacterial effect on *E. coli*, but they did not report any different effect on *S. aureus* [24]. While iron addition had no discernible impact, our investigation demonstrated that Ag addition was especially effective against *E. coli*, and Fe additions of 3 and 5% were shown to considerably boost the antibacterial efficacy against *S. aureus* by 87.8% and 61.7%, respectively (*p* < 0.05).

## 4. Conclusions

This study provides the first comprehensive investigation into the effects of Fe additions on the microstructure, mechanical properties, corrosion behavior, and biological response of Zn-3Ag alloys produced by powder metallurgy. The novel Zn-3Ag-xFe (x = 0, 1, 3, 5 wt.%) alloys were synthesized using mechanical alloying followed by a press-and-sinter consolidation process. The key findings from this work are as follows:

1. Zn-Ag-Fe alloys produced through mechanical alloying exhibited intermetallic phases of AgZn_3_ and Fe_3_Zn_10_, as verified by XRD analysis. SEM/EDX examination indicated that the dimensions and proportion of the intermetallic phase expanded with the increase in alloying content.
2. Prior to sintering, the alloying constituents rendered the material less compressible and more porous. Following sintering, the porosity significantly decreased. The Zn-3Ag-5Fe alloy exhibited 15.35% porosity, whereas the pure Zn demonstrated 11.62% porosity.
3. The hardness increased with the rising concentration of alloying elements due to the formation of hard intermetallic phases. Zn-3Ag-5Fe attained a hardness of 132.8 HV. The Archard equation demonstrated that wear resistance improved with an increase in alloying content.
4. The addition of Ag and Fe increased the nobility (OCP: −986.7 mV → −774.6 mV). The corrosion rate increased from 0.51 to 2.81 mm/year due to the material’s increased porosity and higher intermetallic content. The EIS results indicated that pure Zn exhibited the highest resistivity.
5. All samples demonstrated toxicity at 100% extract concentration, although cell viability surpassed 85% at 12.5% extract concentration. The addition of Fe diminished cell viability due to an elevated corrosion rate. The addition of Ag did not have a substantial cytotoxic effect.
6. The addition of Ag enhanced its efficacy against *E. coli*, but not against *S. aureus*. Increased Fe enhanced the antibacterial action against *S. aureus* by facilitating more degradation.

Considering all of the above conditions, the Zn-3Ag-5Fe alloy is considered the optimum alloy for implants among the alloys produced with its high mechanical properties and acceptable biosafety and antibacterial properties.

## Figures and Tables

**Table 1:** Pre- and post-sintering densities obtained according to Archimedes tests and porosity values according to theoretical density.

Sample	Theoretical Density (g/cm^3^)	Density Before Sintering (g/cm^3^)	Density After Sintering (g/cm^3^)	Porosity Before Sintering (%)	Porosity After Sintering (%)
Zn	7.14	5.89 ± 0.07	6.31 ± 0.02	17.51 ± 0.97	11.62 ± 0.28
Zn-3Ag	7.21	5.76 ± 0.04	6.22 ± 0.06	20.11 ± 0.56	13.73 ± 0.84
Zn-3Ag-1Fe	7.22	5.66 ± 0.08	6.14 ± 0.08	21.61 ± 1.12	14.95 ± 1.10
Zn-3Ag-3Fe	7.23	5.75 ± 0.03	6.18 ± 0.01	20.47 ± 0.42	14.52 ± 0.14
Zn-3Ag-5Fe	7.25	5.62 ± 0.05	6.12 ± 0.04	22.26 ± 0.47	15.35 ± 0.32

**Table 2:** OCP and potentiodynamic polarization results.

Sample	OCP (mV)	E_corr_ (mV)	I_corr_ (µA/cm^2^)	Corrosion Rate (mm/Year)
Zn	−986.7	−1119.2	29.44	0.51
Zn3Ag	−984.0	−1086.1	33.12	0.56
Zn3Ag1Fe	−965.4	−1079.6	51.61	0.88
Zn3Ag3Fe	−957.5	−959.3	154.19	2.63
Zn3Ag5Fe	−774.6	−779.5	164.65	2.81

**Table 3:** Corrosion resistance and matrix resistance results according to EIS tests.

Sample	R_o_ (Ωcm^2^)	R_m_ (Ωcm^2^)	R_total_ (Ωcm^2^)
Zn	183.81	78.24	262.05
Zn3Ag	41.47	188.74	230.21
Zn3Ag1Fe	16.07	183.76	199.83
Zn3Ag3Fe	41.12	117.21	158.33
Zn3Ag5Fe	8.66	101.84	110.5

## Data Availability

The original contributions presented in the study are included in the article; further inquiries can be directed to the corresponding author.

## Supplementary Materials

The following supporting information can be downloaded at https://www.mdpi.com/article/10.3390/jfb16120435/s1, Figure S1: (a) XRD patterns of pure Zn powders after different milling durations, (b) variation in crystallite size and lattice strain of pure Zn powders during milling process; Figure S2: (a) XRD patterns of Zn-3Ag powders after different milling durations, (b) variation in crystallite size and lattice strain of pure Zn-3Ag powders during milling process; Figure S3: XRD patterns of Zn-3Ag-xFe powders after different milling durations: (a) x = 1, (b) x = 3, and (c) x = 5; Figure S4: Variation in crystallite size and lattice strain of Zn-3Ag-xFe powders during milling process: (a) x = 1, (b) x = 3, and (c) x = 5; Figure S5: SEM images of pure Zn powders after different milling durations; Figure S6: SEM images of pure Zn-3Ag powders after different milling durations; Figure S7: SEM images of pure Zn-3Ag-1Fe powders after different milling durations; Figure S8: SEM images of pure Zn-3Ag-3Fe powders after different milling durations; Figure S9: SEM images of pure Zn-3Ag-5Fe powders after different milling durations; Figure S10: Powder size distribution of 20 h milled powder alloys: (a) pure Zn, (b) Zn-3Ag, (c) Zn-3Ag-1Fe, (d) Zn-3Ag-3Fe, and (e) Zn-3Ag-5Fe; Figure S11: Vickers hardness indentations for: (a) pure Zn, (b) Zn-3Ag, (c) Zn-3Ag-1Fe, (d) Zn3Ag-3Fe, and (e) Zn-3Ag-5Fe; Figure S12: Wear track profiles for the consolidated pure Zn and Zn-3Ag-xFe alloys after pin-on disk testing; Figure S13: Live/dead staining of MC3T3-E1 cells. Green fluorescence indicates live cells, white a Live/dead staining of MC3T3-E1 cells. Green fluorescence indicates live cells, white arrows indicate and red dots indicate dead cells.

## Abbreviations

The following abbreviations are used in this manuscript:

MA	Mechanical alloying
SEM	Scanning electron microscopy
EDX	Energy-dispersive X-ray spectroscopy
XRD	X-ray diffraction
FWHM	Width at half maximum
MRI	Magnetic resonance imaging
HV	Vickers microhardness
HBSS	Hank’s balanced salt solution
SCE	Saturated calomel electrode
OCP	Open circuit potential
α-MEM	alpha-minimum essential medium
FBS	Fetal bovine serum
CCK-8	Cell counting kit-8
OD	Optical density
PBS	phosphate-buffer solution
7-AAD	7-aminoactinomycin D
CFU	Colony-forming unit
ANOVA	Analysis of variance
P	Porosity
EIS	Electrochemical impedance spectroscopy
E_corr	Corrosion potential
i_corr	Current density
Rs	Solution resistance
Ro	Corrosion product resistance
Rm	Charge transfer resistance
Qo	Capacitance of the corrosion products
Qm	Capacitance between the zinc alloy and the solution
LC50	Llethal concentration

## References

[1] Makena I.M., Shongwe M.B. (2024). Effects of Porosity on the Corrosion Behaviour of PM-Fabricated Titanium Foams for Biomedical Applications. Int. J. Electrochem. Sci..

[2] Yng Y., Chan K.F., Abd Samad M.I., Ramlee M.H., Yaakob Y., Tanemura M., Mohd Yusop M.Z. (2025). Powder Metallurgy of Zn–Mn/CNF Biodegradable Composites: Role of Ball Milling and Sintering on Material Performance. Mater. Chem. Phys..

[3] Zheng Y.F., Gu X.N., Witte F. (2014). Biodegradable Metals. Mater. Sci. Eng. R Rep..

[4] Yuan K., Deng C., Tan L., Wang X., Yan W., Dai X., Du R., Zheng Y., Zhang H., Wang G. (2024). Structural and Temporal Dynamics Analysis of Zinc-Based Biomaterials: History, Research Hotspots and Emerging Trends. Bioact. Mater..

[5] Katarivas Levy G., Goldman J., Aghion E. (2017). The Prospects of Zinc as a Structural Material for Biodegradable Implants—A Review Paper. Metals.

[6] Cimpoesu N., Paleu V., Panaghie C., Roman A.-M., Cazac A.M., Cioca L.-I., Bejinariu C., Lupescu S.C., Axinte M., Popa M. (2024). Mechanical Properties and Wear Resistance of Biodegradable ZnMgY Alloy. Metals.

[7] Qin Y., Wen P., Voshage M., Chen Y., Schückler P.G., Jauer L., Xia D., Guo H., Zheng Y., Schleifenbaum J.H. (2019). Additive Manufacturing of Biodegradable Zn-XWE43 Porous Scaffolds: Formation Quality, Microstructure and Mechanical Properties. Mater. Des..

[8] Shuai C., Zhao Y., Li C., Deng Y., Zhao Z., Gao C. (2023). Supersaturated Solid Solution Enhanced Biodegradable Zn-Mn Alloys Prepared by Mechanical Alloying and Selective Laser Melting. J. Alloys Compd..

[9] Yang H., Jia B., Zhang Z., Qu X., Li G., Lin W., Zhu D., Dai K., Zheng Y. (2020). Alloying Design of Biodegradable Zinc as Promising Bone Implants for Load-Bearing Applications. Nat. Commun..

[10] Li H.F., Xie X.H., Zheng Y.F., Cong Y., Zhou F.Y., Qiu K.J., Wang X., Chen S.H., Huang L., Tian L. (2015). Development of Biodegradable Zn-1X Binary Alloys with Nutrient Alloying Elements Mg, Ca and Sr. Sci. Rep..

[11] Sun J.-L., Feng Y., Shi Z.-Z., Xue Z., Cao M., Yao S.-L., Li Z., Wang L.-N. (2023). Biodegradable Zn-0.5Li Alloy Rib Plate: Processing Procedure Development and in Vitro Performance Evaluation. J. Mater. Sci. Technol..

[12] Jia B., Yang H., Han Y., Zhang Z., Qu X., Zhuang Y., Wu Q., Zheng Y., Dai K. (2020). In Vitro and in Vivo Studies of Zn-Mn Biodegradable Metals Designed for Orthopedic Applications. Acta Biomater..

[13] Du S., Shen Y., Zheng Y., Cheng Y., Xu X., Chen D., Xia D. (2023). Systematic in Vitro and in Vivo Study on Biodegradable Binary Zn-0.2 At% Rare Earth Alloys (Zn-RE: Sc, Y, La–Nd, Sm–Lu). Bioact. Mater..

[14] Xiao X., Liu E., Shao J., Ge S. (2021). Advances on Biodegradable Zinc-Silver-Based Alloys for Biomedical Applications. J. Appl. Biomater. Funct. Mater..

[15] Güner A.T., Meran C. (2020). Ortopedik implantlarda kullanılan biyomalzemeler TT—Biomaterials used in orthopedic implants. Pamukkale Üniversitesi Mühendislik Bilim. Derg..

[16] Ramirez–Ledesma A.L., Roncagliolo-Barrera P., Alvarez–Perez M.A., Juarez–Islas J.A., Paternoster C., Copes F., Mantovani D. (2023). Introducing Novel Bioabsorbable Zn–Ag–Mg Alloys Intended for Cardiovascular Applications. Mater. Today Commun..

[17] Shuai C., Xue L., Gao C., Yang Y., Peng S., Zhang Y. (2018). Selective Laser Melting of Zn–Ag Alloys for Bone Repair: Microstructure, Mechanical Properties and Degradation Behaviour. Virtual Phys. Prototyp..

[18] Mostaed E., Sikora-Jasinska M., Ardakani M.S., Mostaed A., Reaney I.M., Goldman J., Drelich J.W. (2020). Towards Revealing Key Factors in Mechanical Instability of Bioabsorbable Zn-Based Alloys for Intended Vascular Stenting. Acta Biomater..

[19] Xue P., Ma M., Li Y., Li X., Yuan J., Shi G., Wang K., Zhang K. (2020). Microstructure, Mechanical Properties, and in Vitro Corrosion Behavior of Biodegradable Zn-1Fe-XMg Alloy. Materials.

[20] Li S., Wang X., Ren J., Liu C., Hu Y., Yang Y. (2023). Microstructure, Mechanical Property and Corrosion Behavior of Biomedical Zn-Fe Alloy Prepared by Low-Temperature Sintering. J. Alloys Compd..

[21] Králová Z.O., Gorejová R., Oriňaková R., Petráková M., Oriňak A., Kupková M., Hrubovčáková M., Sopčák T., Baláž M., Maskaľová I. (2021). Biodegradable Zinc-Iron Alloys: Complex Study of Corrosion Behavior, Mechanical Properties and Hemocompatibility. Prog. Nat. Sci. Mater. Int..

[22] Tang L., Chen H., Zhu X., Zubair M., Sun T., Yang L., Lu X., Song Z. (2025). Enhancing Mechanical and Biodegradation Properties of Zn-0.5Fe Alloys Through Rotary Forging. Materials.

[23] Zhang J., Zhu X., Guo P., Zhang Y., Xu D., Pang Y., Song Z., Yang L. (2024). Effects of Li Addition on the Properties of Biodegradable Zn–Fe–Li Alloy: Microstructure, Mechanical Properties, Corrosion Behavior, and Cytocompatibility. Mater. Today Commun..

[24] Su Y., Fu J., Lee W., Du S., Qin Y.-X., Zheng Y., Wang Y., Zhu D. (2022). Improved Mechanical, Degradation, and Biological Performances of Zn–Fe Alloys as Bioresorbable Implants. Bioact. Mater..

[25] Avior O., Ben Ghedalia-Peled N., Ron T., Vago R., Aghion E. (2020). The Effect of Ca on In Vitro Behavior of Biodegradable Zn-Fe Alloy in Simulated Physiological Environments. Metals.

[26] Kafri A., Ovadia S., Yosafovich-Doitch G., Aghion E. (2018). In Vivo Performances of Pure Zn and Zn-Fe Alloy as Biodegradable Implants. J. Mater. Sci. Mater. Med..

[27] Xu Y., Xu Y., Zhang W., Li M., Wendel H.-P., Geis-Gerstorfer J., Li P., Wan G., Xu S., Hu T. (2022). Biodegradable Zn-Cu-Fe Alloy as a Promising Material for Craniomaxillofacial Implants: An in Vitro Investigation into Degradation Behavior, Cytotoxicity, and Hemocompatibility. Front. Chem..

[28] Liu A., Qin Y., Dai J., Song F., Tian Y., Zheng Y., Wen P. (2024). Fabrication and Performance of Zinc-Based Biodegradable Metals: From Conventional Processes to Laser Powder Bed Fusion. Bioact. Mater..

[29] Chu X., Fu Z., Liu Y., Dai Y., Wang J., Song J., Dong Z., Yan Y., Yu K. (2024). Mechanical Properties, Biodegradable Behavior and Biocompatibility of Zn-0.5Ti Alloy Membranes Produced by Powder Metallurgy for Guided Bone Regeneration. Mater. Lett..

[30] Kolawole M.Y., Aweda J.O., Abdulkareem S., Bello S.A. (2020). Biodegradable Zinc Alloys and Composites for Biomedical Application: An Overview of Processing Routes and Possible Future Work. Eur. J. Mater. Sci. Eng..

[31] Čákyová V., Gorejová R., Kupková M., Sopčák T., Strečková M., Fáberová M., Özaltin K., Džupon M., Oriňaková R. (2025). Study of Zn-Ag Alloys Prepared via Powder Metallurgy. SSRN.

[32] Kumar R., Katyal P. (2022). Effects of Alloying Elements on Performance of Biodegradable Magnesium Alloy. Mater. Today Proc..

[33] Rai A., Rai P., Kumar V., Singh N.K., Singh V.K. (2021). Effect of Sintering Temperature on the Physico-Mechanical Behavior of SiC Reinforced Zinc-Magnesium Based Composite. Met. Mater. Int..

[34] Mohamad Rodzi S.N.H., Zuhailawati H., Dhindaw B.K. (2019). Mechanical and Degradation Behaviour of Biodegradable Magnesium–Zinc/Hydroxyapatite Composite with Different Powder Mixing Techniques. J. Magnes. Alloy.

[35] (2023). Metallic Materials—Vickers Hardness Test—Part 1: Test Method.

[36] Dag I.E., Kilic F., Panigrahi M., Avar B. (2025). Microstructure, Mechanical Properties, and Antibacterial Performance of Novel Fe-Mn-Zn Nanocrystalline Alloys Produced by Mechanical Alloying. Adv. Eng. Mater..

[37] Dağ İ.E., Avar B., Panigrahi M., Gündeş A., Şimşek T., Rajendrachari S. (2025). Evaluation of the Microstructural, Mechanical, and Corrosion Behavior of BN-, B4C-, and FeB-Added Distaloy AE Composites Produced by High-Energy Ball Milling and Hot-Pressing. Metallogr. Microstruct. Anal..

[38] Suryanarayana C. (2001). Mechanical Alloying and Milling. Prog. Mater. Sci..

[39] Sumner M., Harison J., Elda S. (2018). Pearson New International Edition. British Library Cataloguing-in-Publication Data.

[40] Suryanarayana C., Norton M.G. (1998). X-Rays and Diffraction BT. X-Ray Diffraction: A Practical Approach.

[41] Liu Z., Qiu D., Wang F., Taylor J.A., Zhang M. (2014). The Grain Refining Mechanism of Cast Zinc through Silver Inoculation. Acta Mater..

[42] Sikora-Jasinska M., Mostaed E., Mostaed A., Beanland R., Mantovani D., Vedani M. (2017). Fabrication, Mechanical Properties and in Vitro Degradation Behavior of Newly Developed ZnAg Alloys for Degradable Implant Applications. Mater. Sci. Eng. C.

[43] Valdez S., Campillo B., Pérez R., Martínez L., García H A. (2008). Synthesis and Microstructural Characterization of Al–Mg Alloy–SiC Particle Composite. Mater. Lett..

[44] Xie Y., Zhao L., Zhang Z., Wang X., Wang R., Cui C. (2018). Fabrication and Properties of Porous Zn-Ag Alloy Scaffolds as Biodegradable Materials. Mater. Chem. Phys..

[45] Yilmazer H., Basit S., Sen A., Yilmazer Y., Dalbayrak B., Arisan E.D., Arisan S., Islamgaliev R.K., Dikici B. (2023). A Comprehensive Study on Microstructure, in-Vitro Biodegradability, Bacterial Sensitivity, and Cellular Interactions of Novel Ternary Zn-Cu-XAg Alloys for Urological Applications. J. Alloys Compd..

[46] Akinwekomi A.D., Akhtar F. (2023). Tunability of Mechanical and Biodegradation Properties of Zinc-Based Biomaterial with Calcium Micronutrient Alloying. J. Mech. Behav. Biomed. Mater..

[47] Youness R.A., Taha M.A. (2024). Tuning Biodegradability, Bone-Bonding Capacity, and Wear Resistance of Zinc-30% Magnesium Intermetallic Alloy for Use in Load-Bearing Bone Applications. Sci. Rep..

[48] Yan Y., Cao H., Kang Y., Yu K., Xiao T., Luo J., Deng Y., Fang H., Xiong H., Dai Y. (2017). Effects of Zn Concentration and Heat Treatment on the Microstructure, Mechanical Properties and Corrosion Behavior of as-Extruded Mg-Zn Alloys Produced by Powder Metallurgy. J. Alloys Compd..

[49] Jara-Chávez G., Amaro-Villeda A., Campillo-Illanes B., Ramírez-Argáez M., González-Rivera C. (2024). Effect of Ag and Cu Content on the Properties of Zn-Ag-Cu-0.05Mg Alloys. Metals.

[50] Xiao X., Wang B., Liu E., Liu H., Liu L., Xu W., Ge S., Shao J. (2023). Investigation of Zinc-Silver Alloys as Biodegradable Metals for Orthopedic Applications. J. Mater. Res. Technol..

[51] Zhou L., Zhang J., Tian Y., Wang J., He D. (2024). Comparison of the Relationship between Hardness and Wear Resistance of Polycrystalline Diamond and Cubic Boron Nitride. J. Am. Ceram. Soc..

[52] Tong X., Wang H., Zhu L., Han Y., Wang K., Li Y., Ma J., Lin J., Wen C., Huang S. (2022). A Biodegradable in Situ Zn–Mg2Ge Composite for Bone-Implant Applications. Acta Biomater..

[53] Li H., Huang J., Zhang P., Zhang Q. (2021). Investigation on Tribological Behaviors of Biodegradable Pure Zn and Zn-X (Li, Cu, Ge) Binary Alloys. J. Mater. Sci. Mater. Med..

[54] Krüger J.T., Hoyer K.-P., Schaper M. (2022). Bioresorbable AgCe and AgCeLa Alloys for Adapted Fe-Based Implants. Mater. Lett..

[55] Schinhammer M., Hänzi A.C., Löffler J.F., Uggowitzer P.J. (2010). Design Strategy for Biodegradable Fe-Based Alloys for Medical Applications. Acta Biomater..

[56] Guan Z., Linsley C.S., Pan S., DeBenedetto C., Liu J., Wu B.M., Li X. (2020). Highly Ductile Zn-2Fe-WC Nanocomposite as Biodegradable Material. Metall. Mater. Trans. A.

[57] Gabay N., Ron T., Vago R., Shirizly A., Aghion E. (2021). Evaluating the Prospects of Ti-Base Lattice Infiltrated with Biodegradable Zn–2%Fe Alloy as a Structural Material for Osseointegrated Implants—In Vitro Study. Materials.

[58] Nová K., Novák P., Průša F., Kopeček J., Čech J. (2019). Synthesis of Intermetallics in Fe-Al-Si System by Mechanical Alloying. Metals.

[59] Gaylan Y., Avar B., Panigrahi M., Aygün B., Karabulut A. (2023). Effect of the B4C Content on Microstructure, Microhardness, Corrosion, and Neutron Shielding Properties of Al–B4C Composites. Ceram. Int..

[60] Tong X., Dong Y., Han Y., Zhou R., Zhu L., Zhang D., Dai Y., Shen X., Li Y., Wen C. (2024). A Biodegradable Zn-5Gd Alloy with Biomechanical Compatibility, Cytocompatibility, Antibacterial Ability, and in Vitro and in Vivo Osteogenesis for Orthopedic Applications. Acta Biomater..

[61] (2009). Part 5: Tests for In Vitro Cytotoxicity.

[62] Shi Z.-Z., Yu J., Liu X.-F., Zhang H.-J., Zhang D.-W., Yin Y.-X., Wang L.-N. (2019). Effects of Ag, Cu or Ca Addition on Microstructure and Comprehensive Properties of Biodegradable Zn-0.8Mn Alloy. Mater. Sci. Eng. C.

[63] Chen C., Yue R., Zhang J., Huang H., Niu J., Yuan G. (2020). Biodegradable Zn-1.5Cu-1.5Ag Alloy with Anti-Aging Ability and Strain Hardening Behavior for Cardiovascular Stents. Mater. Sci. Eng. C.

[64] Wang X., Liu A., Zhang Z., Hao D., Liang Y., Dai J., Jin X., Deng H., Zhao Y., Wen P. (2024). Additively Manufactured Zn-2Mg Alloy Porous Scaffolds with Customizable Biodegradable Performance and Enhanced Osteogenic Ability. Adv. Sci..

