# Supplementary material for: Effect of Iron on the Microstructure, Mechanical Properties, Corrosion Behavior, and Biocompatibility of Mechanically Alloyed Zn-3Ag Biodegradable Alloys
Source: J Funct Biomater. 2025 Nov 25;16(12):435. doi: 10.3390/jfb16120435 (PMC12733797; doi:10.3390/jfb16120435)
Supplement: Supplementary file 1 [file jfb-16-00435-s001.zip › jfb-3883720-supplementary.pdf]

Supplementary Materials

Effect of Iron on the Microstructure, Mechanical Properties, Corrosion Behavior, and Biocompatibility of Mechanically Alloyed Zn-3Ag Biodegradable Alloys

Ilker Emin Dag[1,2], *Ebru Erdal*[3], *Mohsen Mhadhbi*[4] *and Baris Avar*[1,2,*]

[1]*Department of Nanotechnology Engineering, Zonguldak Bülent Ecevit University, Zonguldak 67100, Türkiye*

[2]*Department of Metallurgical and Materials Engineering, Zonguldak Bülent Ecevit University, Zonguldak 67100, Türkiye*

[3]*Advanced Technologies Application and Research Center, Ankara Yıldırım Beyazıt University, Ankara 06031, Türkiye*

[4]*Laboratory of Useful Materials, National Institute of Research and Physicochemical Analysis, Technopole Sidi Thabet, 2020 Ariana, Tunisia*

[*]*Correspondence: barisavar@beun.edu.tr*

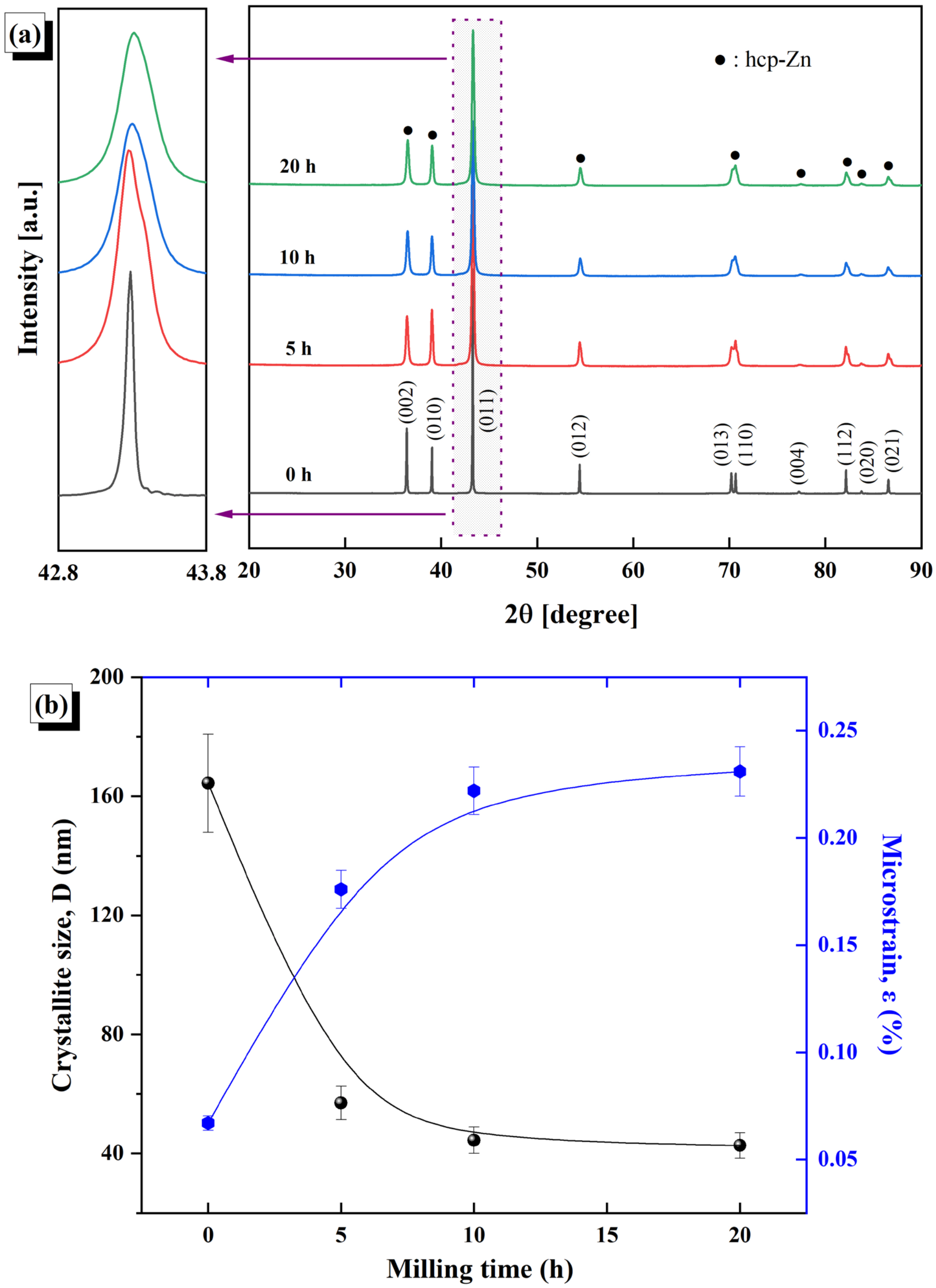

Figure S1. (a) XRD patterns of pure Zn powders after different milling durations, (b) variation in crystallite size and lattice strain of pure Zn powders during milling process.

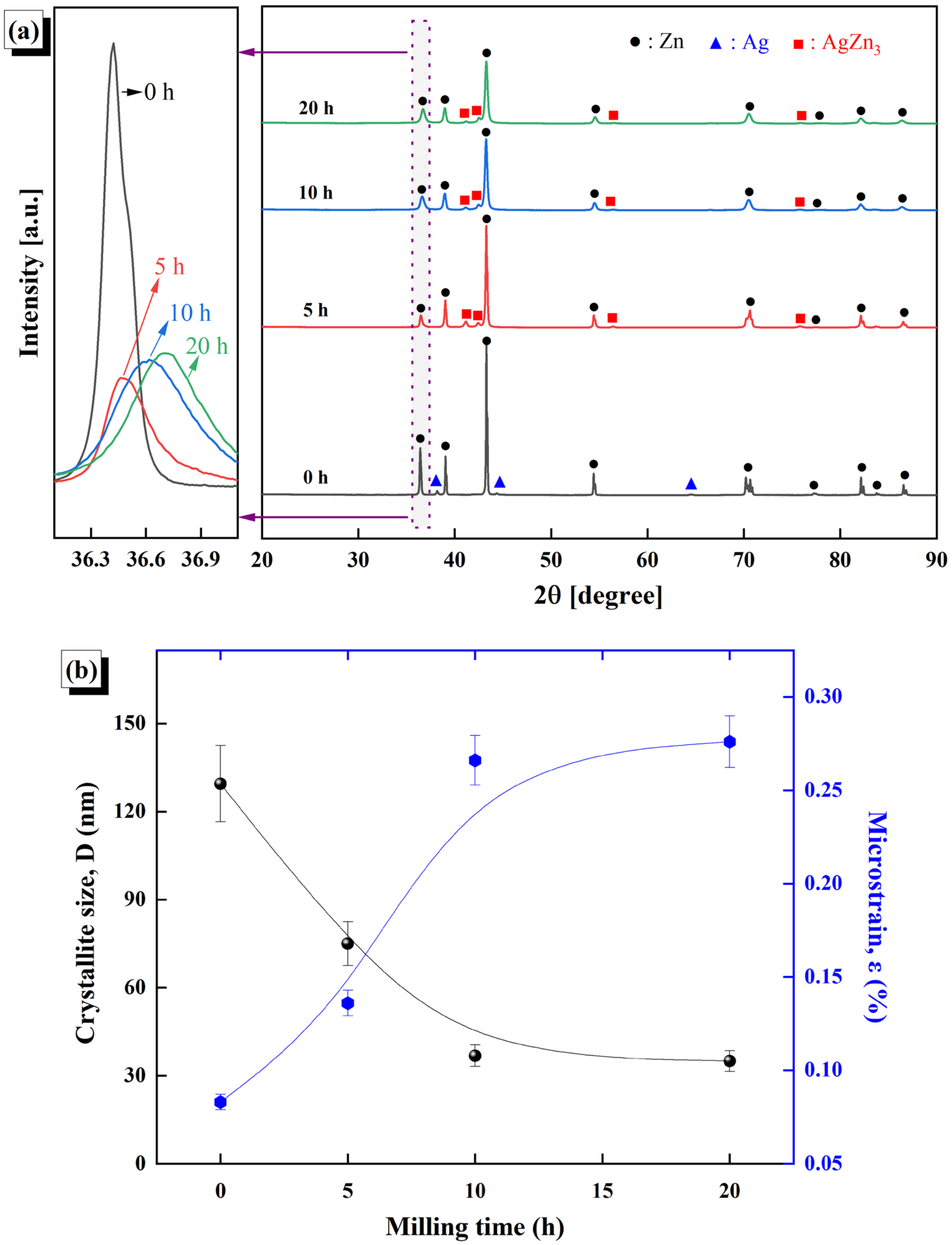

Figure S2. (a) XRD patterns of Zn-3Ag powders after different milling durations, (b) variation in crystallite size and lattice strain of pure Zn-3Ag powders during milling process.

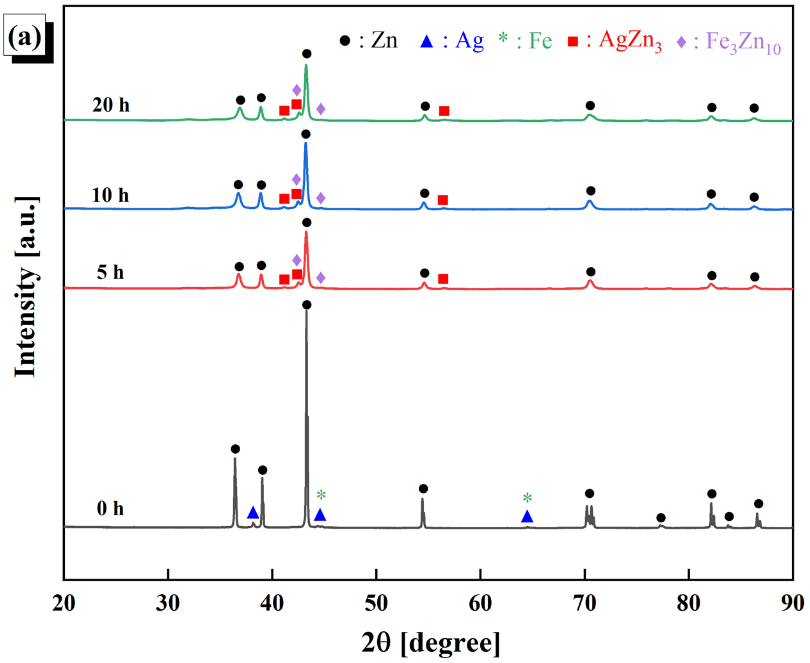
(a)
● : Zn ▲ : Ag * : Fe ■ : AgZn3 ♦ : Fe3Zn10
20 h
10 h
5 h
0 h
Intensity [a.u.]
20 30 40 50 60 70 80 90
2θ [degree]

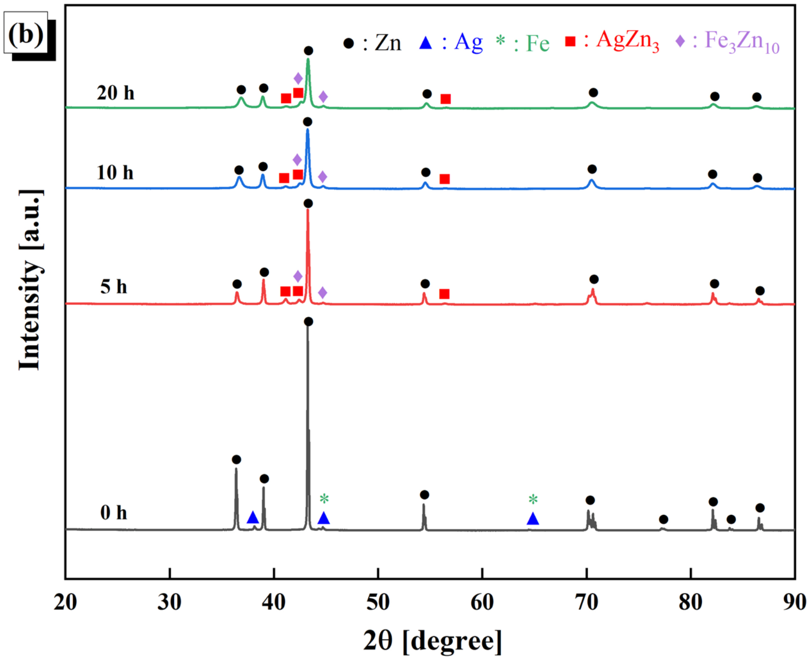
(b)
● : Zn ▲ : Ag * : Fe ■ : AgZn3 ♦ : Fe3Zn10
20 h
10 h
5 h
0 h
Intensity [a.u.]
20 30 40 50 60 70 80 90
2θ [degree]

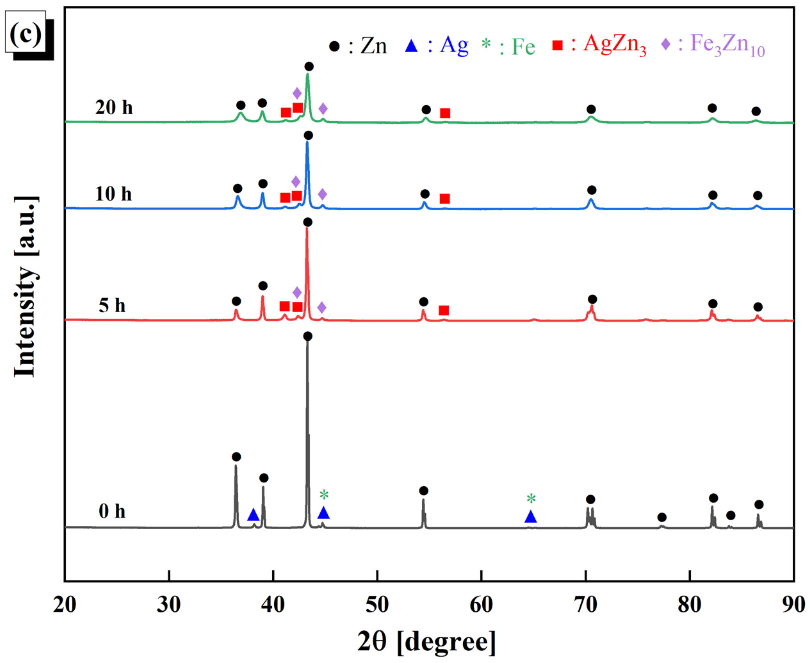

Figure S3. XRD patterns of Zn-3Ag-xFe powders after different milling durations: (a) x=1, (b) x=3, and (c) x=5.

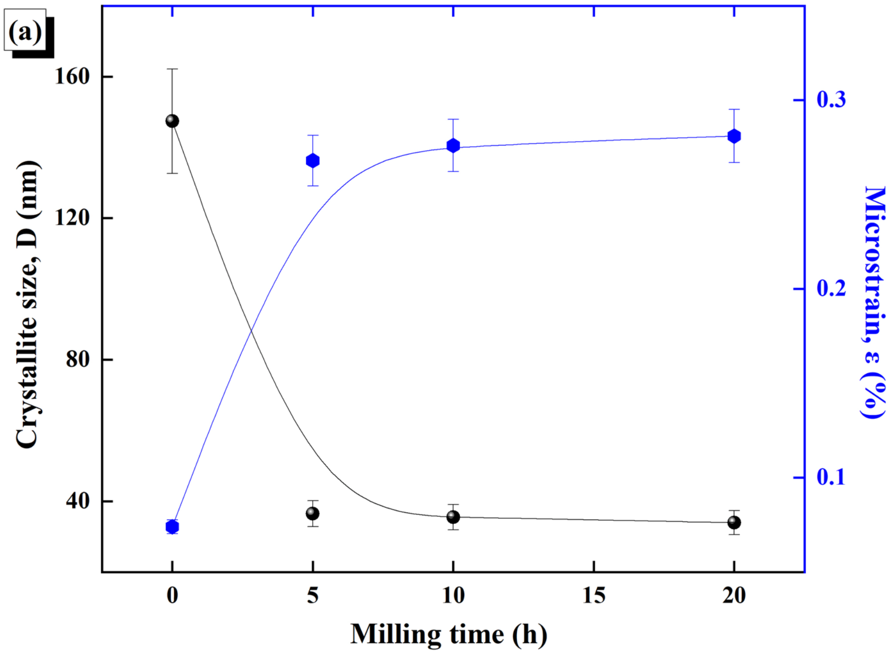

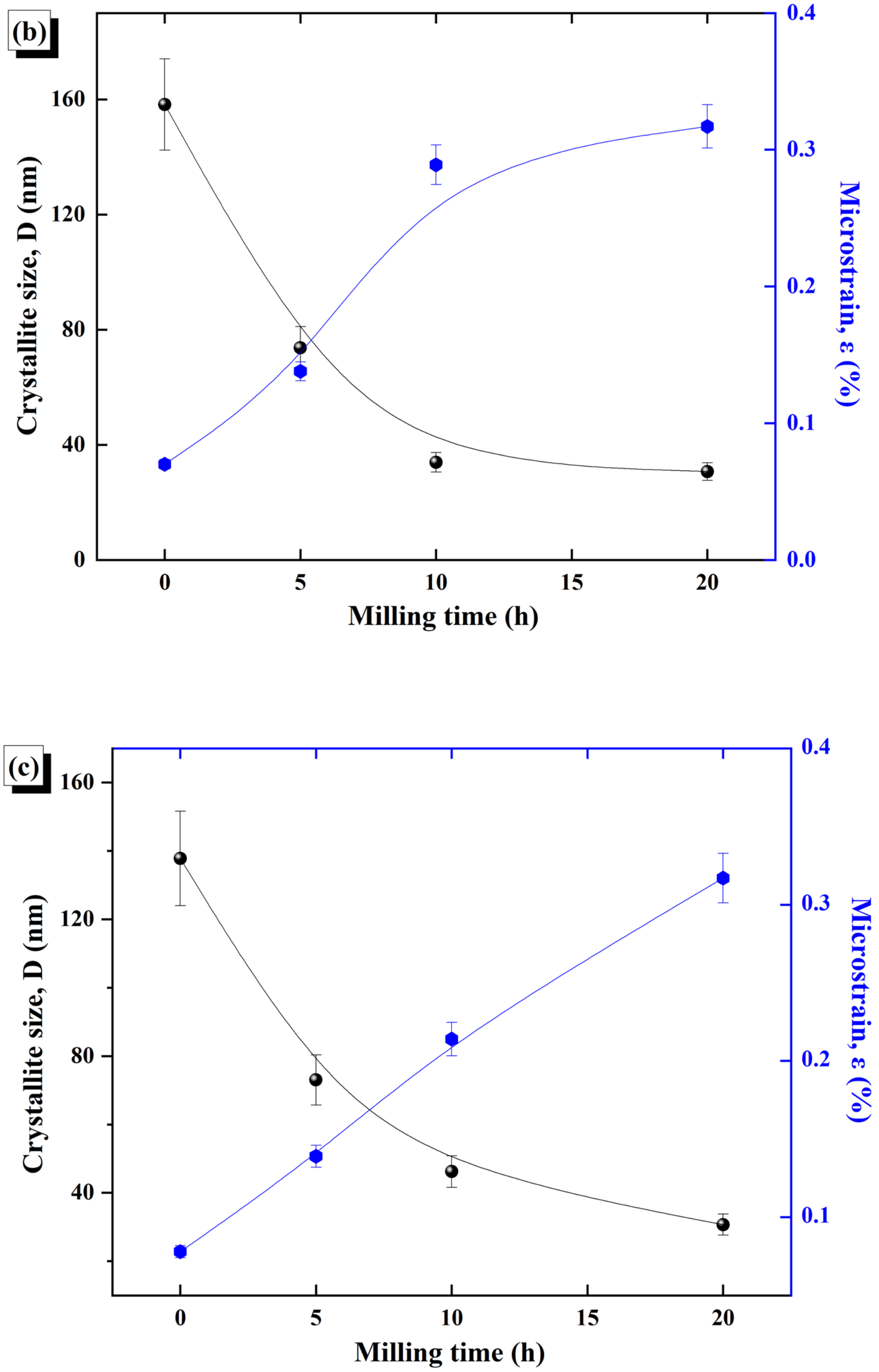

Figure S4. Variation in crystallite size and lattice strain of Zn-3Ag-xFe powders during milling process: (a) x=1, (b) x=3, and (c) x=5.

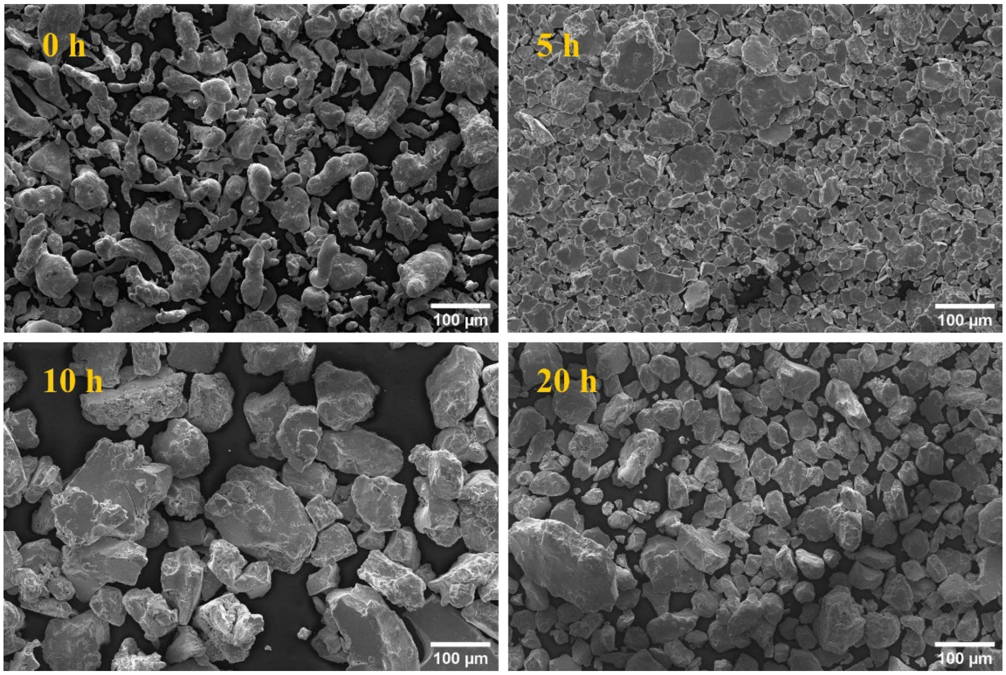

Figure S5. SEM images of pure Zn powders after different milling durations.

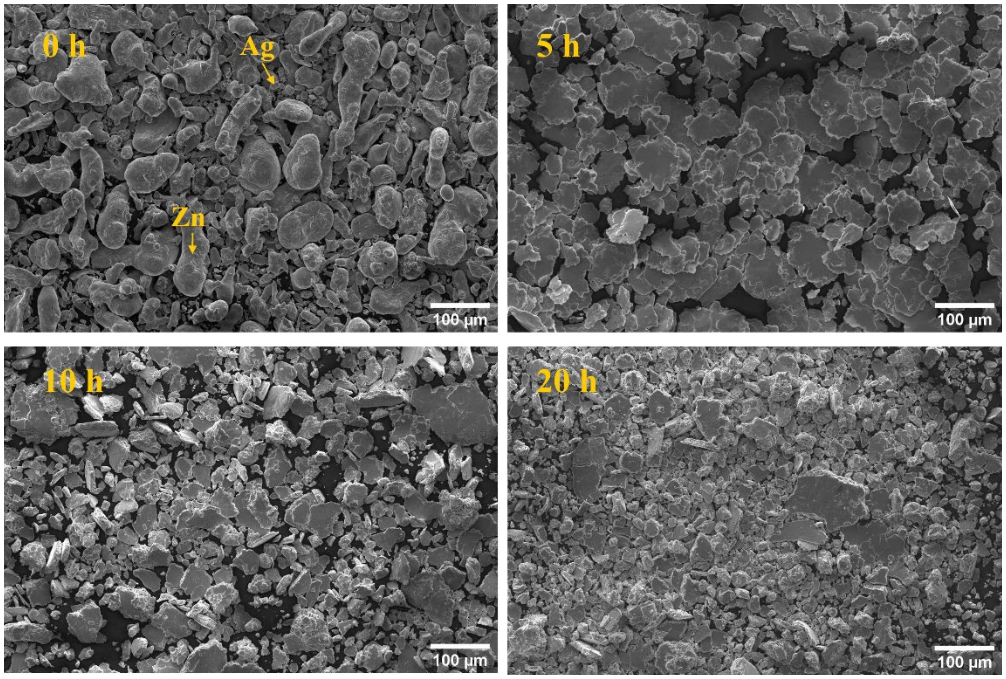

Figure S6. SEM images of pure Zn-3Ag powders after different milling durations.

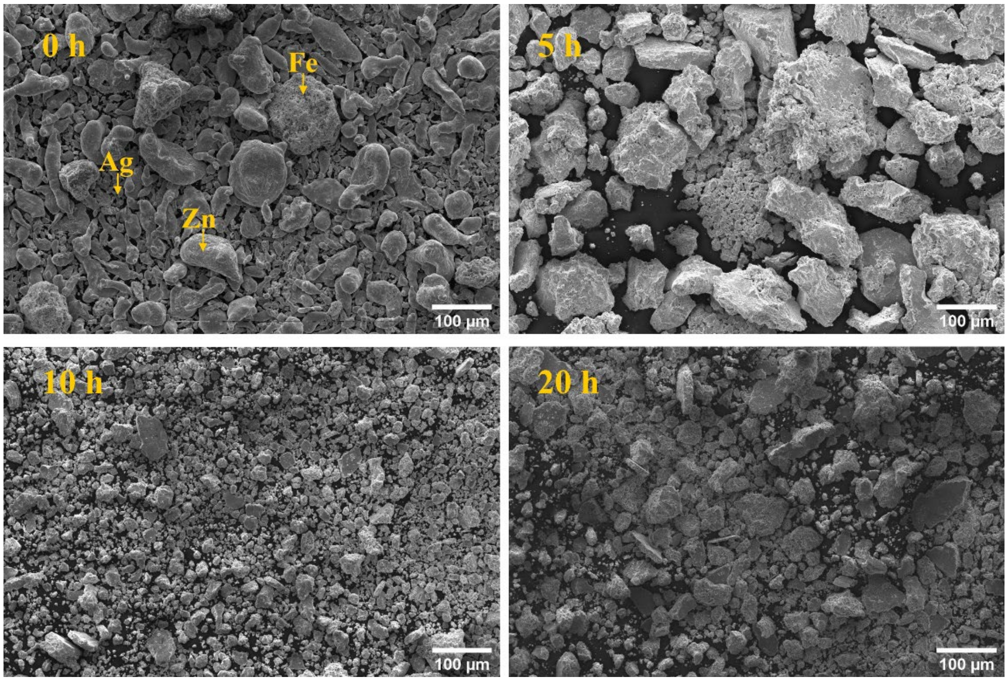

Figure S7. SEM images of pure Zn-3Ag-1Fe powders after different milling durations.

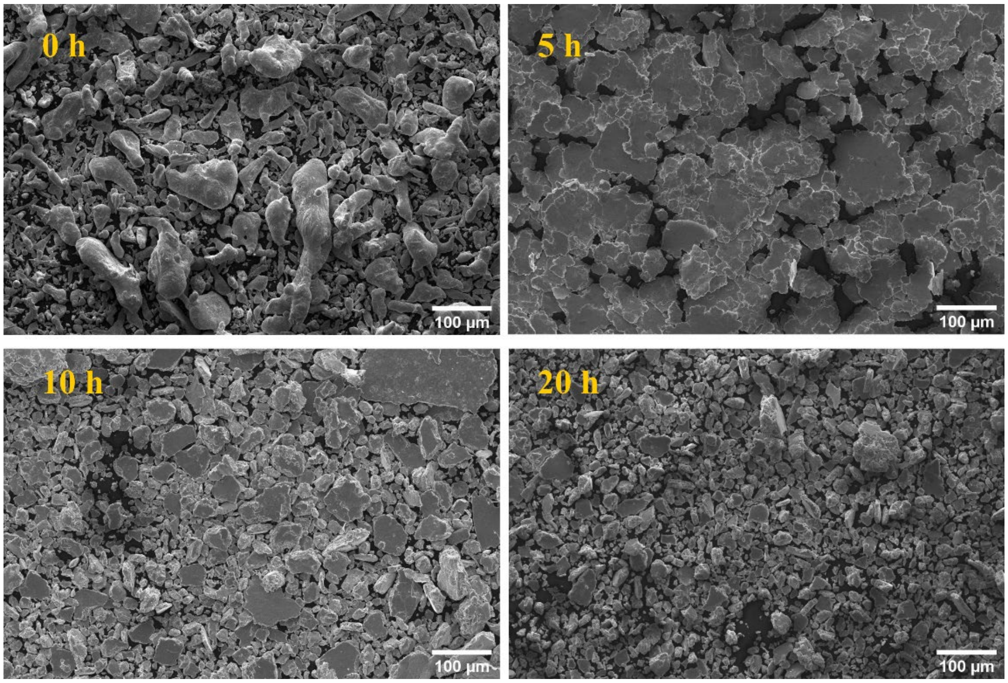

Figure S8. SEM images of pure Zn-3Ag-3Fe powders after different milling durations.

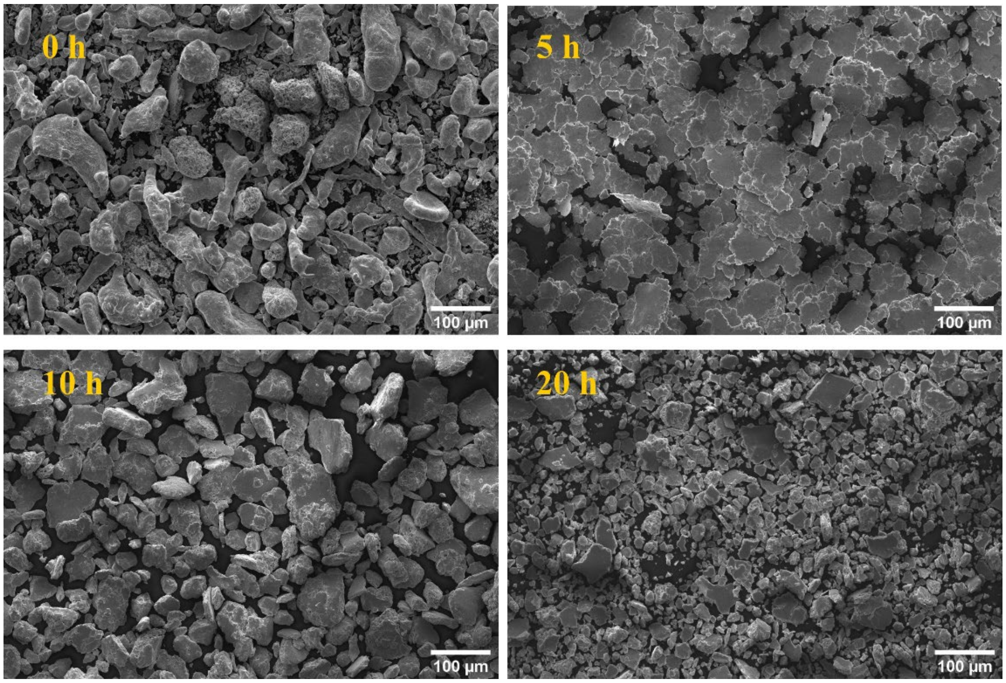

Figure S9. SEM images of pure Zn-3Ag-5Fe powders after different milling durations.

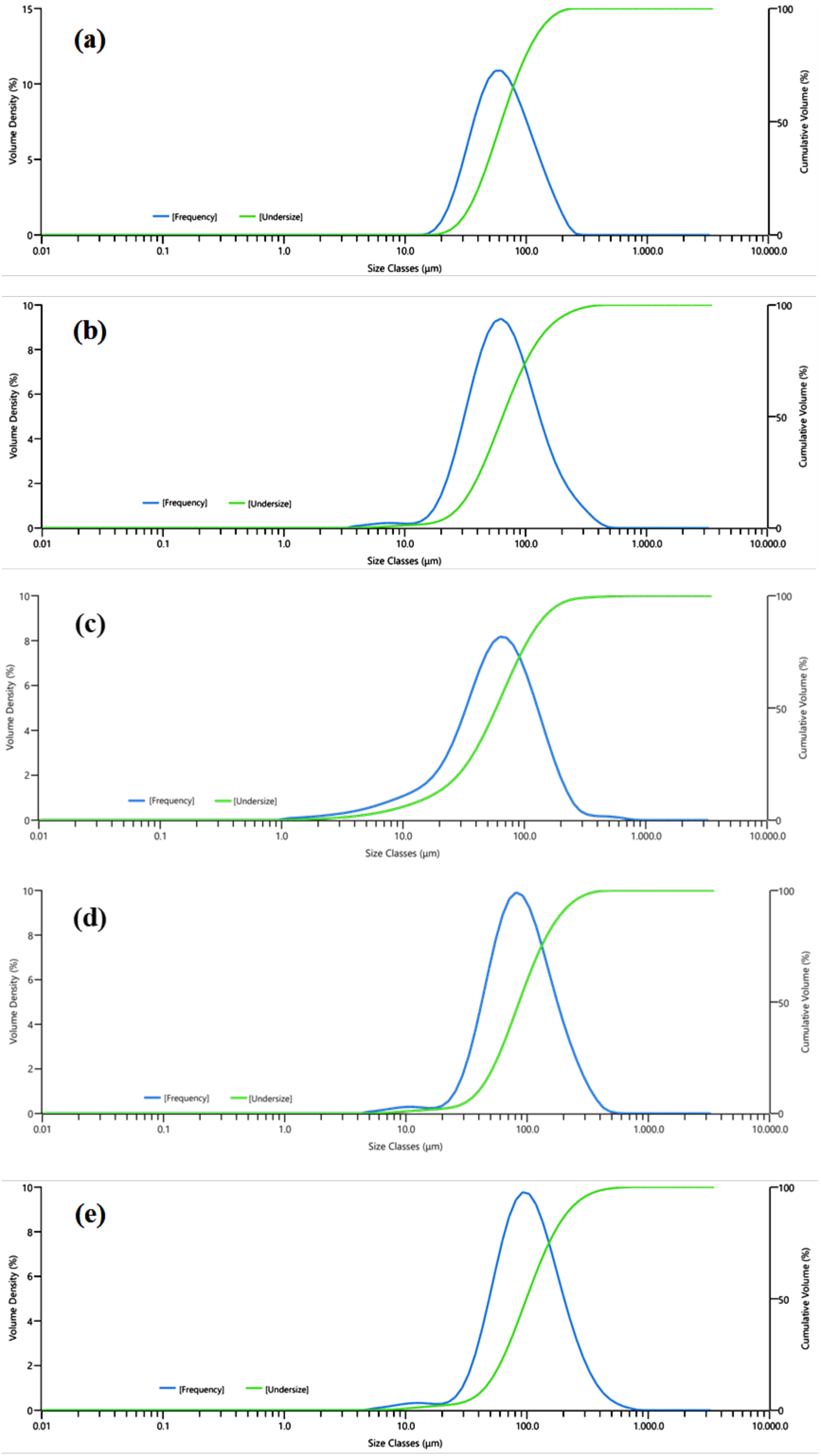

Figure S10. Powder size distribution of 20 h milled powder alloys: (a) pure Zn, (b) Zn-3Ag, (c) Zn-3Ag-1Fe, (d) Zn-3Ag-3Fe, and (e) Zn-3Ag-5Fe.

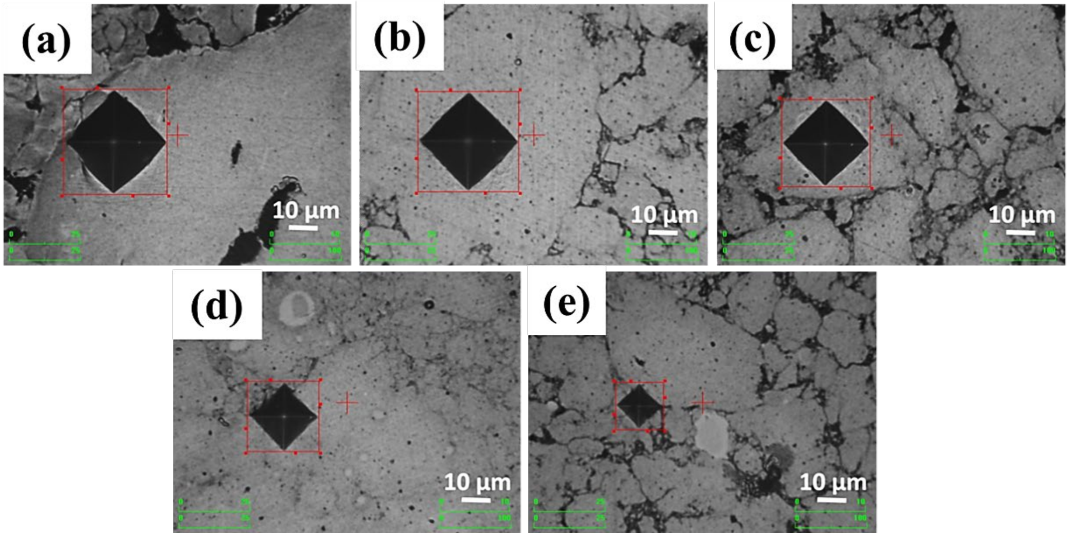

Figure S11. Vickers hardness indentations for: (a) pure Zn, (b) Zn-3Ag, (c) Zn-3Ag-1Fe, (d) Zn-3Ag-3Fe, and (e) Zn-3Ag-5Fe.

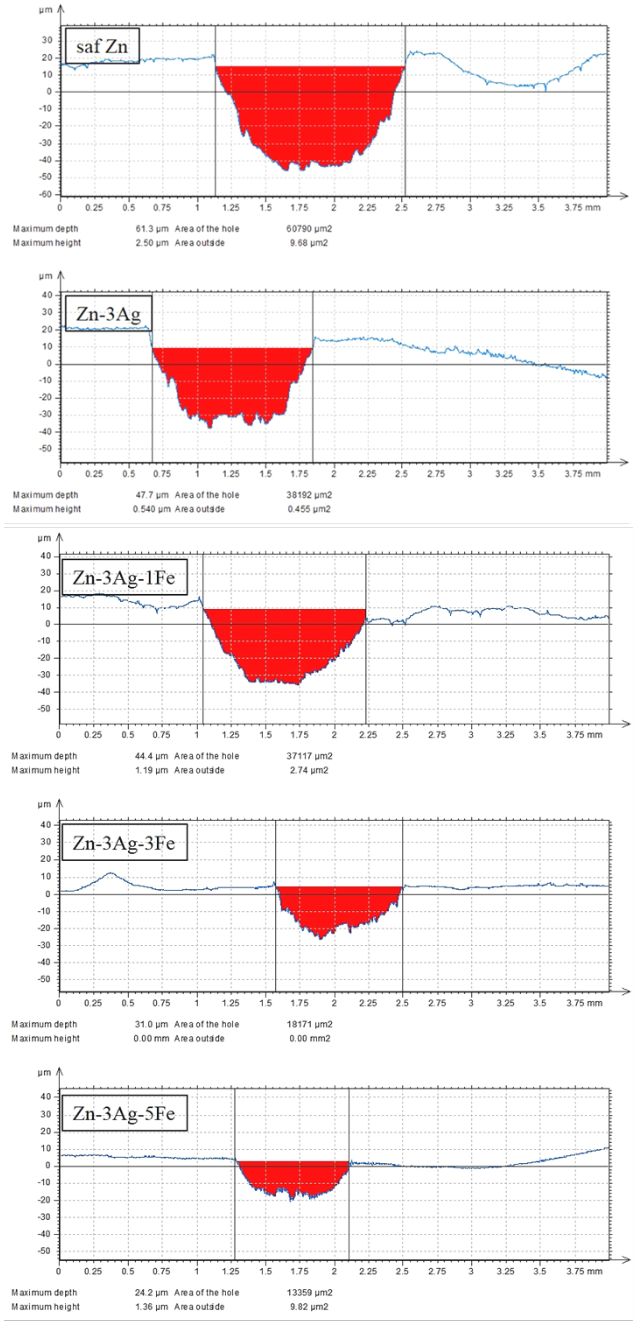

Figure S12. Wear track profiles for the consolidated pure Zn and Zn-3Ag-xFe alloys after pin-on-disk testing.

	Calcein AM	7 ADD	Merge
**Positive Control**	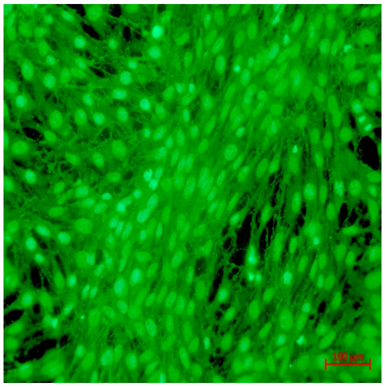	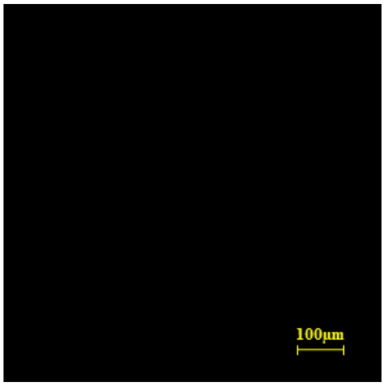	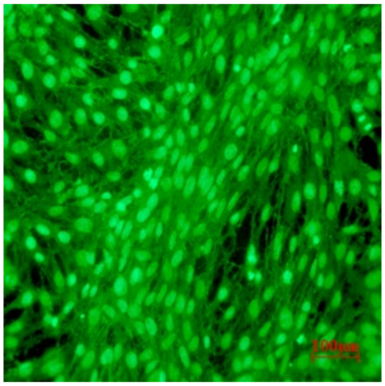
**Negative Control**	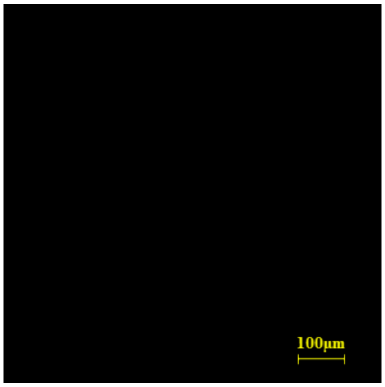	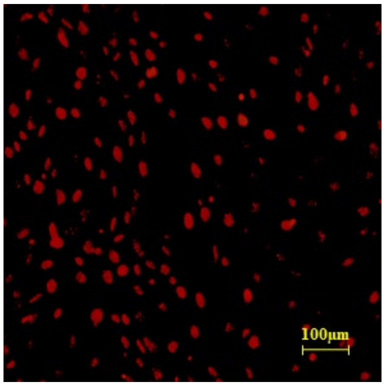	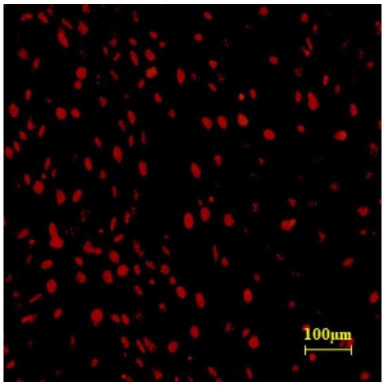

100% Extract	Calcein AM	7 ADD	Merge
**Zn**	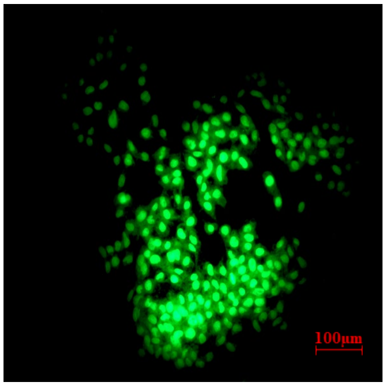	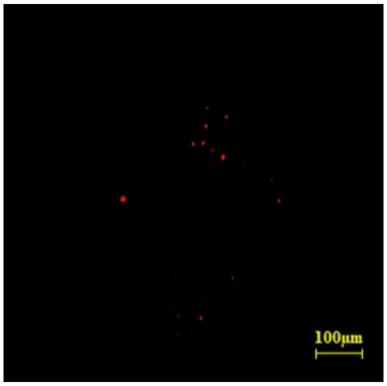	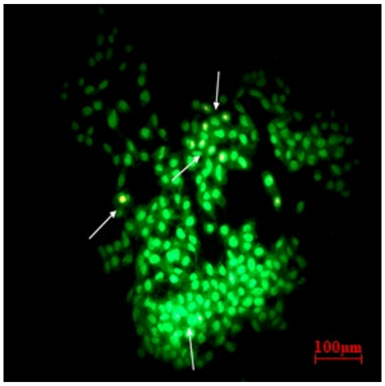

**Zn-3Ag**	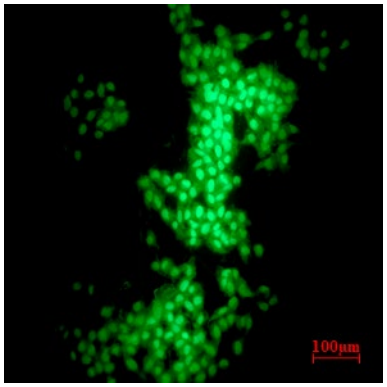	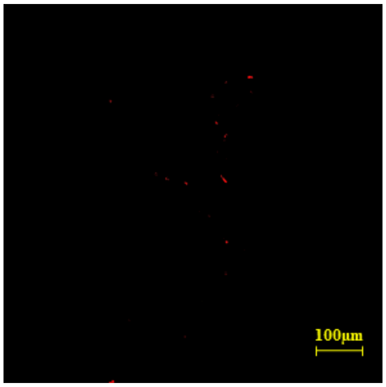	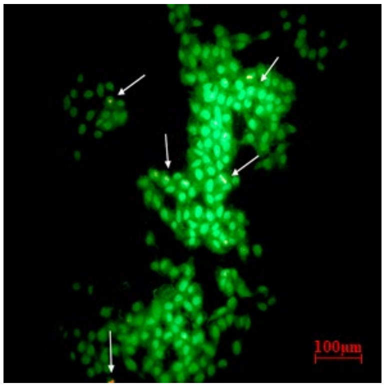
**Zn-3Ag-1Fe**	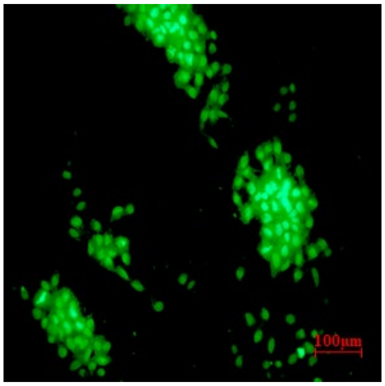	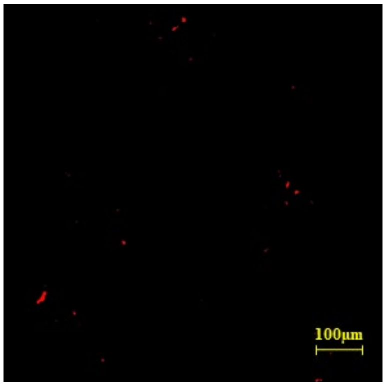	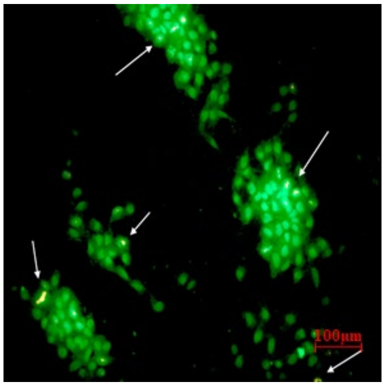
**Zn-3Ag-3Fe**	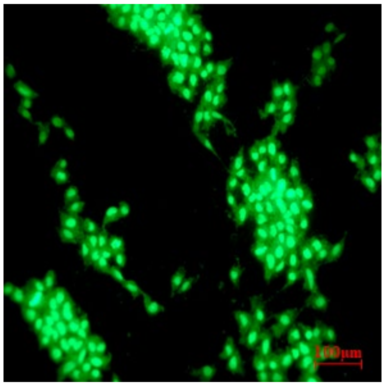	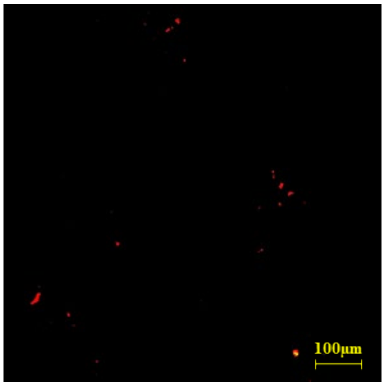	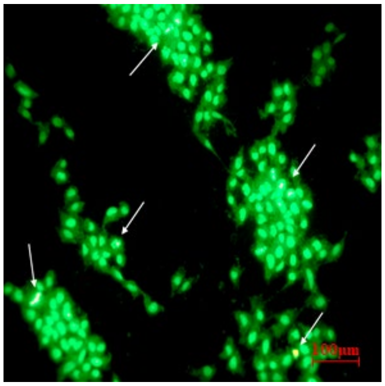
**Zn-3Ag-5Fe**	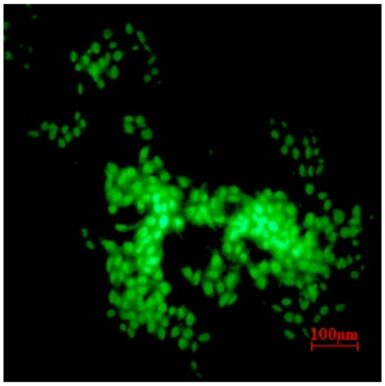	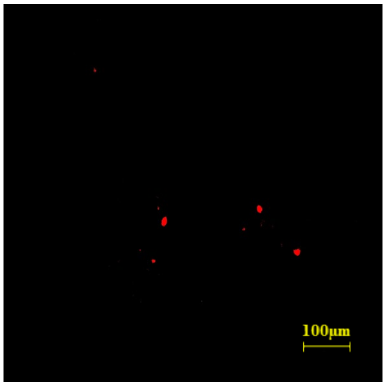	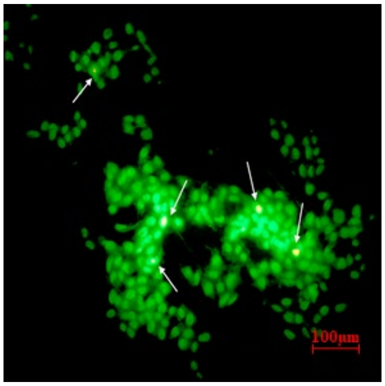

12.5% Extract	Calcein AM	7 ADD	Merge
**Zn**	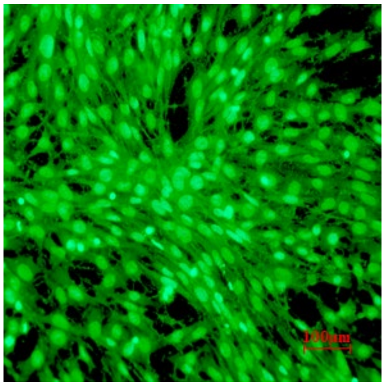	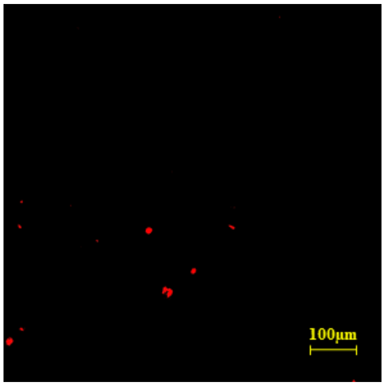	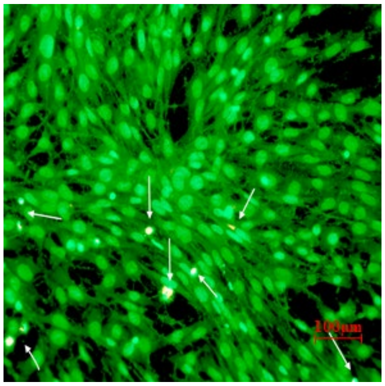
**Zn-3Ag**	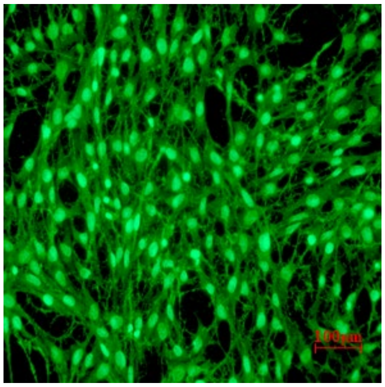	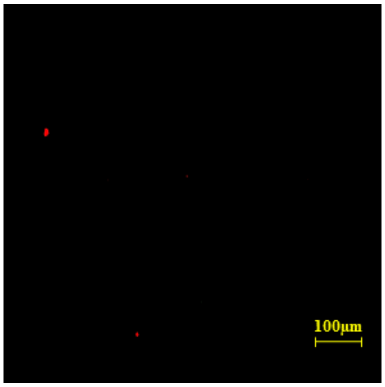	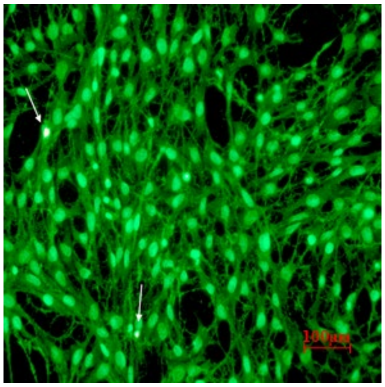
**Zn-3Ag-1Fe**	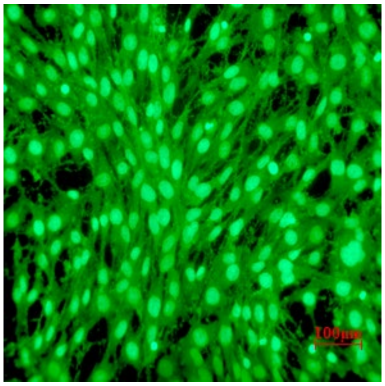	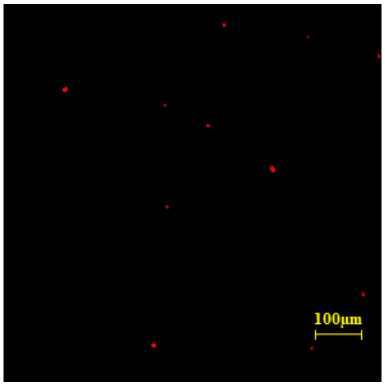	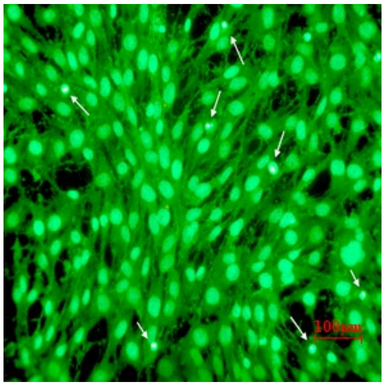
**Zn-3Ag-3Fe**	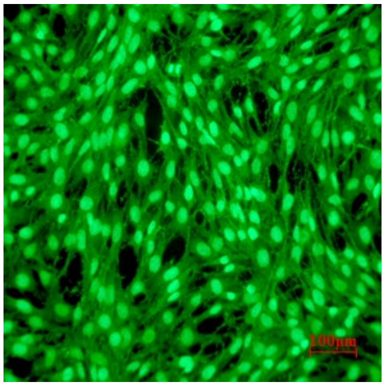	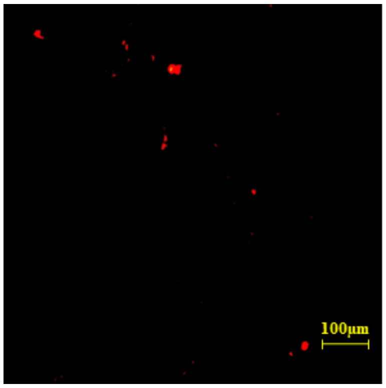	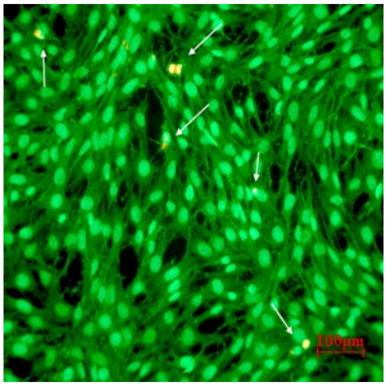

**Zn-3Ag-5Fe**	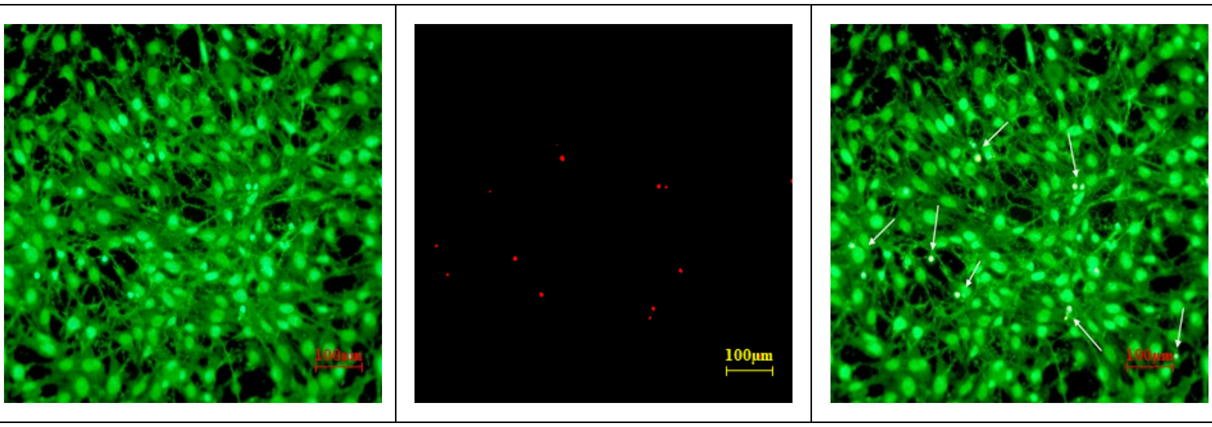

Figure S13. Live/dead staining of MC3T3-E1 cells. Green fluorescence indicates live cells, white a Live/dead staining of MC3T3-E1 cells. Green fluorescence indicates live cells, white arrows indicate, and red dots indicate dead cells.
